# Characterization of GM3 Gangliosides in Human Milk throughout Lactation: Insights from the Analysis with the Use of Reversed-Phase Liquid Chromatography Coupled to Quadrupole Time-Of-Flight Mass Spectrometry

**DOI:** 10.1021/acs.jafc.3c04489

**Published:** 2023-11-13

**Authors:** Weronika Hewelt-Belka, Michał Młynarczyk, Dorota Garwolińska, Agata Kot-Wasik

**Affiliations:** Department of Analytical Chemistry, Faculty of Chemistry, Gdańsk University of Technology, 80-233 Gdańsk, Poland

**Keywords:** gangliosides, human milk, ganglioside composition, GM3, LC-MS

## Abstract

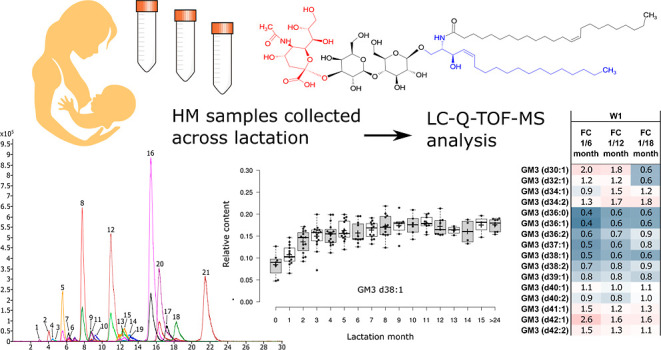

Gangliosides are complex lipids found in human milk that play important structural and biological functions. In this study, we utilized reversed-phase liquid chromatography coupled to quadrupole time-of-flight mass spectrometry to evaluate the molecular distribution of GM3 in human milk samples collected at distinct lactation stages, ranging from colostrum to advanced lactation samples. Throughout lactation, GM3 d40:1 emerged as the most abundant GM3 species, except in colostrum, where GM3 d42:2 prevailed. The relative content of GM3 species containing very long N-fatty acyl (N-FA) substituents with >22 carbon atoms decreased, while the content of GM3 species containing 14:0, 18:0, 18:1, and 20:0 N-FA substituents increased in the later months of lactation. These findings highlight the divergence of GM3 profiles across the lactation period. Moreover, considerable interindividual variance was observed among the analyzed samples. The assessment of the GM3 profiles contributes to our understanding of the dynamic composition of human milk.

## Introduction

Human milk (HM) contains a variety of chemical compounds that serve as nutrients or biologically active compounds (growth factors, defense agents, or enzymes). Therefore, it is considered the most valuable source of nutrition for newborns as well as older children along with the complementary food.^1^ Gangliosides (GAs) are complex lipids composed of a ceramide (Cer) and an oligosaccharide that contains one or more sialic acid (SA) residues such as *N*-acetylneuraminic acid (Neu5Ac). The structural diversity of gangliosides is due to variation in the oligosaccharide and ceramide parts, such as different sequences of monosaccharides as well as different lengths and saturation levels of the sphingoid base and N-fatty acyl (N-FA) substituents. GAs are components of almost all human tissue and their composition is tissue-specific, with the highest relative content in the brain (mostly in the neuronal cell membranes in the synaptic area).^2^ In human milk, GM3 and GD3 are the most dominant gangliosides^3−5^ and are located exclusively in the human milk fat globule membrane (MFGM).^6^ It is assumed that the gangliosides found in HM may play a role in brain development,^2^ intestinal protection,^7,8^ and the inhibition of enterotoxins from *Escherichia coli* and *Vibrio cholera*.^9,10^ It was demonstrated that supplementation of infant formula with a ganglioside-enriched dairy fraction has beneficial impacts on cognitive development in infants aged from 0 to 6 months.^11^

The total ganglioside content in human milk changes in the course of lactation,^4,5,12−14^ as well as the content of GM3 and GD3, with GM3 content increasing and GD3 decreasing across the lactation.^5,12−14^ Although the dynamics of the total content of GM3 and GD3 in human milk during lactation have been well studied, information about the changes in the molecular distribution of GA species in the literature is scarce. It was previously reported that the fatty acid as well as long-chain base composition of human milk gangliosides changes throughout lactation.^15,16^ However, there is a lack of data about the GM3 distribution in advanced lactation (HM after 1 year postpartum). The use of liquid chromatography coupled with high-resolution mass spectrometry (LC-HRMS) allows for class identification and evaluation of the ganglioside structure.^13^ This includes the length and level of unsaturation of the ceramide moiety. Therefore, LC-HRMS comprises a valuable tool in ganglioside composition investigation that can fill a knowledge gap about variation in a ganglioside ceramide composition across lactation.

Since early nutrition plays a crucial role in a child’s development and as we progress in our understanding of the role of gangliosides in infant development, further research is needed.^17^ Filling the knowledge gap about the dynamics of the GM3 molecular composition throughout the lactation is necessary to further determine the impact of these components on infant health, growth, and development, as well as to provide suitable nutrition for all infants and move from standardized nutritional protocols to tailored, individualized nutrition for infants.^18^

In this study, we aimed at expanding knowledge about longitudinal dynamics of GM3 molecular composition in human milk throughout the lactation by analyzing human milk samples collected in different months of lactation. We employed reversed-phase liquid chromatography coupled with quadrupole time-of-flight mass spectrometry (RP-LC-Q-TOF-MS) to separate and detect GM3 species. The composition of GM3 was examined and characterized not only with respect to the average composition of GM3 species between time collection points but also within individual mothers. GM3 composition was evaluated in terms of the relative distribution of GM3 species differing in the ceramide moiety structure. To the best of authors’ knowledge, this is the first report where GM3 composition was analyzed in samples collected in advanced lactation, even after 24 months of lactation.

## Materials and Methods

### Chemicals

Methanol (LC–MS-grade) and chloroform (HPLC-grade) were purchased from Merck (Darmstadt, Germany). Ammonium formate (99.9% purity) was purchased from Sigma-Aldrich (St. Louis, MO, USA). The ultrapure water used for the aqueous solutions was produced by an HLP5 system (Hydrolab, Wislina, Poland). The GM3 standard from bovine milk was purchased from Avanti Lipids (Birmingham, AL., USA).

### Human Milk Samples

Human milk samples were collected as previously described.^19^ Briefly, human milk samples were donated by healthy female volunteers (Pomeranian Voivodship, Poland), who had delivered healthy full-term neonates and met the criteria of inclusion to the study (inclusion and exclusion criteria for the study are given in Supporting Information). Written informed consent was obtained from each participant. The milk samples were collected in the evening, with the use of an electronic breast pump, by the full expression of one breast, according to the standardized collection procedure. Ten mL of the collected HM samples was transferred to a polypropylene laboratory tube, kept frozen at −20 °C before the transport to the laboratory, and stored at −80 °C until analysis in the laboratory.^19^

We received and analyzed 176 samples of human milk, including 161 samples collected by 17 volunteers throughout the lactation period each month (samples collected from women marked as W1 to W17), HM samples collected after 24 months of lactation collected by 6 volunteers (from women W18 to W23) and 6 additional colostrum samples collected by 6 different volunteers. Colostrum samples (except sample W17_0) were obtained at the Obstetric Clinic, University Clinical Centre of the Medical University of Gdańsk. The detailed characteristics of the samples collected by the volunteers are presented in Table S1.

The study was performed as a subproject of “The dynamics of Human Breast Milk composition. Long-term metabolomic analysis of Human Breast Milk”. The ethical approval was obtained from the Human Research Ethics Committee of the Medical University of Gdańsk, Poland (decision no. NKBBN/389/2019, date of approval: eighth of July 2019).

### Ganglioside Extraction

A slight modification was made to the methods previously published to extract gangliosides from human milk samples.^20^ One mL of the milk sample (250 μL of the colostrum sample diluted with deionized water 1/4, *v*/*v*) was transferred to a borosilicate-glass tube with a polytetrafluoroethylene (PTFE) cap. Subsequently, 4220 μL of a chloroform/methanol mixture (1/2, *v*/*v*), 1380 μL of chloroform, and 1380 μL of deionized water were added to the tube. The resulting mixture underwent rigorous mixing for 40 s, followed by shaking for 5 min at 1000 rpm, and centrifugation for 10 min at 4200 rpm. The aqueous phase was carefully transferred to a new glass tube using a Pasteur pipet. The residual organic phase underwent a second round of extraction involving 4220 μL of chloroform/methanol mixture (1/2, *v*/*v*), 1380 μL of chloroform, and 1380 μL of deionized water. This was followed by shaking for 5 min at 1000 rpm and another centrifugation step lasting 10 min. The resultant aqueous phases were combined and subjected to purification using the solid-phase extraction (SPE) technique. The columns (StrataTM-X 33 μm, 30 mg/1 mL, Phenomenex, Torrance, CA, USA) were conditioned with a mixture of MeOH/H_2_O (1/1, *v*/*v*). Then, 7 mL of the aqueous phase was applied and washed with 1 mL of MeOH/H_2_O (5/95, *v*/*v*). Desorption was carried out first with 1 mL of a MeOH/H_2_O (4/1, *v*/*v*) mixture and then with 1 mL of MeOH. The resulting extracts were evaporated to dryness under a stream of nitrogen and stored at −80 °C before the analysis. To prepare the extracts for LC–MS analysis, the residual extract was thawed and dissolved in 100 μL of MeOH. It was vortexed and transferred to a chromatography vial.

### Ganglioside Profiling with the Use of LC-Q-TOF-MS

The analysis of gangliosides in human milk samples was conducted utilizing high-performance liquid chromatography coupled with quadrupole time-of-flight mass spectrometry system (HPLC-MS-Q-TOF). An Agilent 1290 LC system, comprising a binary pump, an online degasser, an autosampler, and a thermostated column compartment, was coupled to a 6540 Q-TOF-MS equipped with a Jet Stream Technology Ion Source (Agilent Technologies, Santa Clara, CA, USA). The separation of GM3 species was achieved using a reverse-phase mode chromatographic column, specifically the Kinetex C8 2.1 × 150 mm, 1.7 μm (Phenomenex, Torrance, CA, USA).

The mobile phase consisted of component A, 5 mM ammonium formate in MeOH/H_2_O, 4/1, *v*/*v* and component B, 5 mM ammonium formate in MeOH. The gradient program was configured as follows: 20 to 30% B in 0–25 min, 30 to 90% B in 25–35 min, followed by equilibration at 20% B for 5 min. The mobile phase flow rate and column temperature were maintained at 0.4 mL/min and 50 °C, respectively. The entire run time was 40 min, with an injection volume set to 5 μL. Data acquisition was performed in the negative ionization mode using the SCAN acquisition mode over the range from 500 to 1700 *m*/*z* in the high-resolution mode (4 GHz). The Q-TOF used in this study provides the resolution and full width at maximum (fwhm) in the *m*/*z* range of detected gangliosides as follows: a resolution of 33,001 and an fwhm of 0.0374 for *m*/*z* 1221.9906 (positive ion mode); a resolution of 31,991 and an fwhm of 0.048 for *m*/*z* 1521. 9741 (positive ion mode); a resolution of 41,875 and an fwhm of 0.025 for *m*/*z* 1033. 9881 (negative ion mode); a resolution of 33,790 and an fwhm of 0.0399 for *m*/*z* 1333.9689 (negative ion mode). The MS analysis was conducted with the following parameters: capillary voltage at 4000 V, fragmentation voltage at 120 V, gas flow at 12 L/min, and drying gas and nebulizer temperature set to 300 °C. MS/MS analyses were conducted in both negative and positive ionization modes, maintaining uniform chromatographic and ion source conditions as in MS analyses. Collision energy was set to 35 and 80 V. The two most abundant peaks were chosen for fragmentation and excluded for the subsequent 0.3 min. MS/MS spectra were acquired within the *m*/*z* range of 50–1700. Ganglioside extracts were injected in a randomized manner.

To ensure LC–MS stability control, a single quality control (QC) sample (GM3 bovine milk standard) was injected between real samples throughout the sequence. The LC–MS batch commenced with the extraction blank and the subsequent injection of five QC samples to stabilize the chromatographic column. Ganglioside extracts were maintained at 10 °C in the autosampler during the batch run.

### Data Treatment and Analysis

The determination of peak areas for the identified gangliosides was accomplished utilizing the Batch Targeted Feature Extraction algorithm integrated into the Agilent MassHunter Workstation Profinder 10.0 (Agilent Technologies, Santa Clara, CA, USA). The algorithm was configured with the following parameters: negative ions, charge carriers—H–, match tolerance of 15 ppm, retention time of 0.3 min, and filtering based on peak height set at 1000 counts. Data preparation for subsequent statistical and chemometric analyses was conducted using Microsoft Excel 2016 software (Microsoft Corporation, Redmond, WA, USA). To calculate the percentage relative amount of GM3, the peak area of each GM3 species was divided by the sum of the peak areas of all detected GM3 species. Filtration of data was carried out based on intensity threshold (peak height > 1000 counts) and frequency criteria (GM3 species were retained in the data set if they were present in 80% of the samples).

Precision was used to validate the extraction method. The GM3 data set was included for comparative analysis if the peak area percentage RSD was less than 30% for all extraction replicates. Two approaches were used to evaluate the extraction precision. The gangliosides were extracted from pooled human milk samples (*n* = 5) and the % RSD of the GM3s peak area was calculated. A further evaluation of precision has been conducted for two concentrations of the bovine milk GM3 standard: 12.5 and 2.5 μg/mL. The bovine milk GM3 standard was added to the pooled human milk sample diluted 50-fold with deionized water. A % RSD of normalized peak area (peak area of each GM3 species divided by the total peak area of all GM3) was calculated for the extraction triplicates. Comparative analysis was conducted on GM3 species that met the criteria for filtration (intensity threshold, frequency, and precision). To assess the variation in the abundance of MS signals and their stability over time, the peak area % RSD of GM3 species in QC samples analyzed throughout the sample batch was evaluated. Chemometric and statistical analyses were performed using Metaboanalyst 5.0. The identification of gangliosides involved an automated search using a custom HM database,^19^ based on accurately measured *m*/*z* values with a Δ5 ppm tolerance, and manual interpretation of the obtained MS/MS spectra from human milk samples in both positive and negative ionization modes. To confirm the identity of GM3 species, the diagnostic ion of 290.0892 *m*/*z* that corresponds to the loss of sialic acid in negative ionization mode was used. HM milk samples collected at several lactation stages (>24, 3, 12, 13, 8, 7, and 15 months) were used for the characterization of ceramide part of GM3, and the structures were proposed based on the presence of the ions: 264.2685 *m*/*z* for d18:1, 266.2842 *m*/*z* for d18:0, 262.2529 *m*/*z* for d18:2, and 236.2372 *m*/*z* for d16:1.

## Results

In this study, we investigated the dynamics of GM3 ganglioside profiles in human milk (HM) samples throughout lactation. We analyzed a total of 176 samples: 161 HM samples were collected by 17 volunteers over several months of lactation, starting from the first month. Additionally, we analyzed an additional 6 colostrum samples and 9 samples collected after 24 months of lactation. Detailed characteristics of the analyzed HM samples are provided in Table S1 of the Supporting Information.

### RP-LC-Q-TOF-MS Analysis of Human Milk GM3

**Figure 1:**
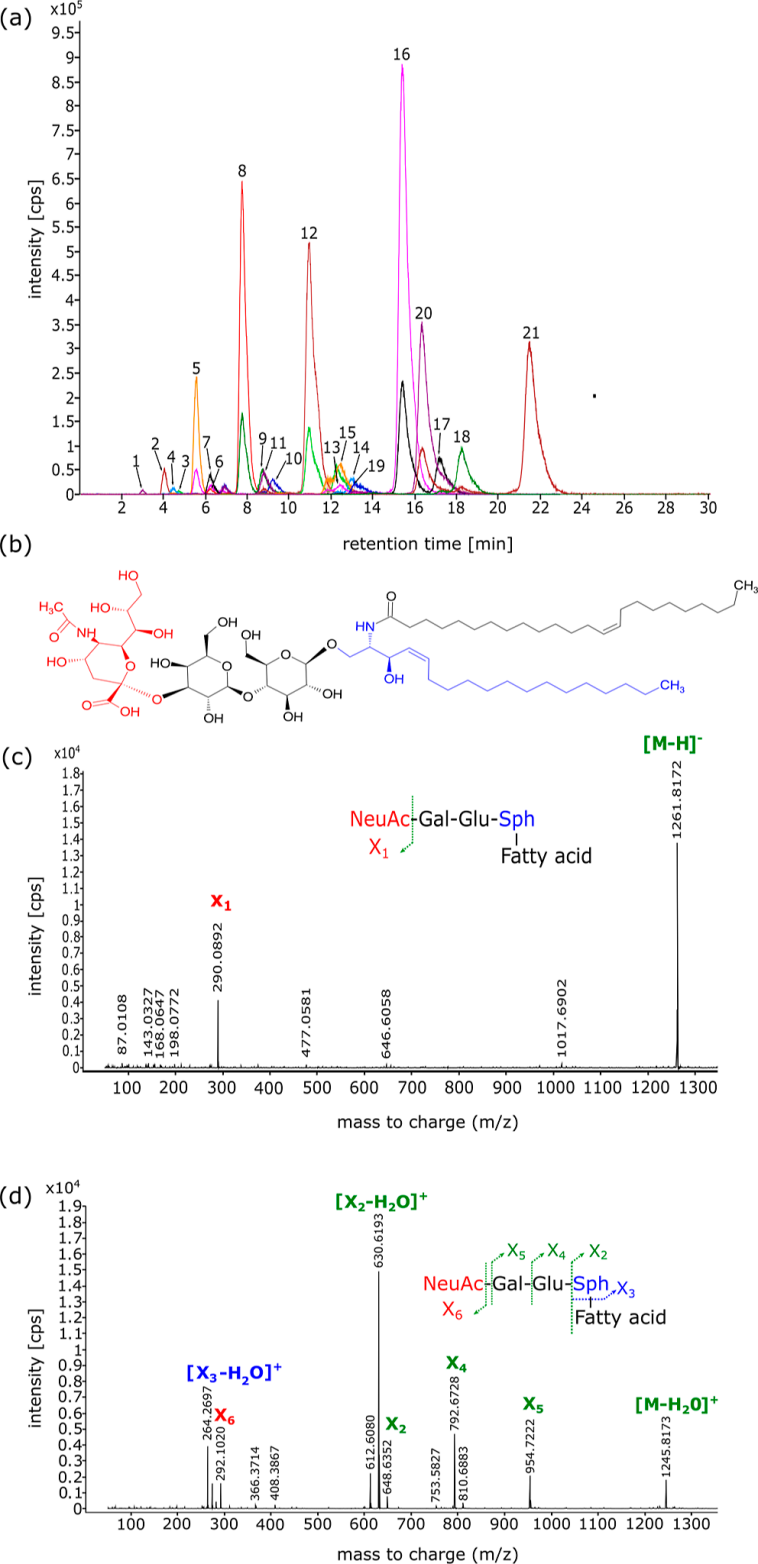
Representative extracted ion chromatogram (EIC) of GM3 gangliosides detected in the human milk sample (a). The structure of GM3 d18:1/24:1 (b). The mass spectra of GM3 d18:1/24:1 obtained in negative (c) and positive (d) ionization mode. 1—GM3 d30:1, 2—GM3 d32:1, 3—GM3 d33:1, 4—GM3 d34:2, 5—GM3 d34:1, 6—GM3 d34:0, 7—GM3 d36:2, 8—GM3 d36:1, 9—GM3 d36:0, 10—GM3 d37:1, 11—GM3 d38:2, 12—GM3 d38:1, 13—GM3 d38:0, 14—GM3 d39:1, 15—GM3 d40:2, 16—GM3 d40:1, 17—GM3 d40:0, 18—GM3 d41:1, 19—GM3 d42:3, and 20—GM3 d42:2, 21—GM3 d42:1.

Ganglioside extracts were analyzed using RP-LC-Q-TOF-MS technique. The utilization of reversed-phase mode chromatography combined with high-resolution mass spectrometry enabled the separation and detection of 39 unique GM3 species, differing in ceramide length and saturation levels. Figure 1a illustrates an exemplary chromatogram of the analyzed gangliosides. The elution of GM3 species was correlated with the length and unsaturation of the ceramide moiety. Specifically, species with longer ceramide chains exhibited longer retention times, while among species with the same ceramide length those with more double bonds demonstrated shorter elution times. This observation aligns with previous studies employing reversed-phase mode chromatography for ganglioside separation.^21^

High-resolution mass spectrometry in two ionization modes was employed to characterize the GM3 species present in the tested HM samples. The experiments for GM3 quantification were conducted in SCAN mode, while the structure elucidation was performed based on MS/MS experiments in the product ion scan. The GM3 species were identified based on an accurate *m*/*z* ratio and confirmed by interpretation of MS/MS spectra. Negative ionization mode was used for the quantification, since GM3 molecules as acidic compounds ionize more effectively by deprotonation, ensuring detection of low abundant species. Also, the diagnostic ion 290.0892 which corresponds to the loss of sialic acid that can be observed on MS/MS spectra obtained in negative ionization mode was used to confirm the identity of GM3 species (see Figure 1c). On the other hand, MS/MS analysis in positive ionization mode allows for the determination of the ceramide moiety structure based on the interpretation of the obtained fragment ions. Hence, the MS/MS experiments in positive ionization mode were performed to propose the structure of the identified GM3 species. The ceramide moiety structure of GM3 species was assessed from MS/MS spectra acquired in positive ionization mode according to the pattern shown in Figure 1d and available literature data.^20^

The results summarized in Table 1 show that the human milk GM3 species contain four distinct long chain bases (LCBs): d18:0, d18:1, d18:2, and d16:1. N-FAs in GM3 species were deduced from the associated LCB, and they showed greater diversity than LCB. Among the N-Fas detected in GM3 species were unsaturated medium-chain Fas (MCFAs) such as 12:0, unsaturated and monosaturated long-chain Fas (LCFAs) such as 18:0 and 18:1, and even very long-chain Fas such as 24:2. The interpretation of MS/MS spectra revealed the presence of GM3 structural isomers with different compositions of LCB and N-fatty acyl, which were not separated chromatographically and coeluted as one peak (e.g*.*, GM3 d42:3, GM3 d40:1, and GM3 d40:2). For instance, on the MS/MS spectrum of GM3 d40:1, fragment ions indicating two possible structures, d18:1/22:0 and d16:1/24:0, were observed: *m*/*z* 264.2684 for d18:2 and *m*/*z* 236.2364 for d16:1 (Figure S1). For some GM3 species, such as GM3 d42:3, GM3 d40:2, and GM3 d38:2, fragment ion *m*/*z* = 262.2529 was observed, suggesting the presence of the sphingoid base with two unsaturated bonds (d18:2) (Supporting Information, Figure S2). Furthermore, chromatographically separated (although not completely) peaks sharing the same *m*/*z* value were observed for GM3 d43:2 and GM3 d41:2, indicating the presence of the different type of GM3 isomers (Supporting Information, Figure S3). However, the MS/MS spectra obtained for these very low abundant GM3 species did not enable the determination of the composition of LCB and N-fatty acids.

**Table 1:** Characteristics of GM3 Species Detected in Human Milk^a^

name	proposed structure	*m*/*z* (ESI−)	sphingoid base fragment *m*/*z* (ESI+)	retention time [min]
GM3 d30:1	d18:1/12:0	1095.6422	264.2685	3.03
GM3 d32:0	na	1125.6841		4.6
GM3 d32:1 iso 1	d18:1/14:0	1123.6722	264.2685	4.06
GM3 d32:1 iso 2	d16:1/16:0	1123.6722	236.2372	4.06
GM3 d33:1	na	1137.6922		4.70
GM3 d34:2	na	1149.6922		4.45
GM3 d34:1 iso 1	d18:1/16:0	1151.7022	264.2685	5.57
GM3 d34:1 iso 2	d16:1/18:0	1151.7022	236.2372	5.57
GM3 d34:0	na	1153.7222		6.31
GM3 d35:1	na	1165.7159		6.5
GM3 d36:2 iso 1	d18:1/18:1	1177.7222	264.2685	6.23
GM3 d36:2 iso 2	d18:2/18:0a	1177.7222	262.2529	6.23
GM3 d36:1 iso 1	d18:1/18:0	1179.7322	264.2685	7.76
GM3 d36:1 iso 2	d16:1/20:0	1179.7322	236.2372	7.76
GM3 d36:0	d18:0/18:0	1181.7522	266.2842	8.76
GM3 d37:1	d18:1/19:0	1193.7522	264.2685	9.21
GM3 d38:2 iso 1	d18:2/20:0	1205.7522	262.2529	8.79
GM3 d38:2 iso 2	d18:1/d20:1	1205.7522	264.2685	8.79
GM3 d38:1 iso 1	d18:1/20	1207.7722	264.2685	10.90
GM3 d38:1 iso 2	d16:1/22:0	1207.7722	236.2372	10.90
GM3 d38:0	d18:0/20:0	1209.7822	266.2842	12.27
GM3 d39:1 iso 1	d18:1/21:0	1221.7822	264.2685	12.95
GM3 d39:1 iso 2	d18:0/21:1	1221.7822	266.2842	12.95
GM3 d40:2 iso 1	d18:2/22:0	1233.7822	262.2529	12.484
GM3 d40:2 iso 2	d18:1/22:1	1233.7822	264.2685	12.484
GM3 d40:2 iso 3	d16:1/24:1	1233.7822	236.2372	12.484
GM3 d40:1 iso 1	d18:1/22:0	1235.8022	264.2685	15.40
GM3 d40:1 iso 2	d16:1/24:0	1235.8022	236.2372	15.40
GM3 d40:0	na	1237.8122		17.18
GM3 41:2 iso 1	na	1247.7925		13.84
GM3 41:2 iso 2	na	1247.7925		14.54
GM3 d41:1	d18:1/23:0	1249.8122	264.2685	18.23
GM3 d42:3 iso 1	d18:1/24:2	1259.8022	264.2685	13.13
GM3 d42:3 iso 2	d18:2/24:1	1259.8022	262.2529	13.13
GM3 d42:2	d18:1/24:1	1261.8122	264.2685	16.34
GM3 d42:1	d18:1/24:0	1263.8322	264.2685	21.49
GM3 d43:2 iso 1		1275.8231		18.3
GM3 d43:2 iso 2		1275.8231		19.2
GM3 d44:1		1291.8551		27.63

a The structure of GM3 species was proposed based on the interpretation of MS/MS spectra acquired in positive ionization mode. GM3 isomers were differentiated based on the sphingoid base fragment ion present in MS/MS spectra (theoretical *m*/*z* are listed in the table). na—MS/MS spectra in positive ion mode was not available due to too low abundance.

### Differences in GM3 Profiles between the HM Samples Collected in Various Lactation Periods

To evaluate the changes in molecular composition of GM3 in HM samples, we analyzed and compared the % relative amount of distinct GM species. A relative amount of each GM3 was calculated based on the following equation: peak area of GM3 divided by the sum of peak areas of all GM3 species. For the purpose of comparative analysis, the signal obtained in negative ionization mode was used due to the higher intensity of the MS signal. After data filtration, 16 GM3 species were included in the comparative analysis (peak height threshold > 1000, % RSD in QC samples < 30%). The results of analytical validation are shown in Table S2.

**Figure 2:**
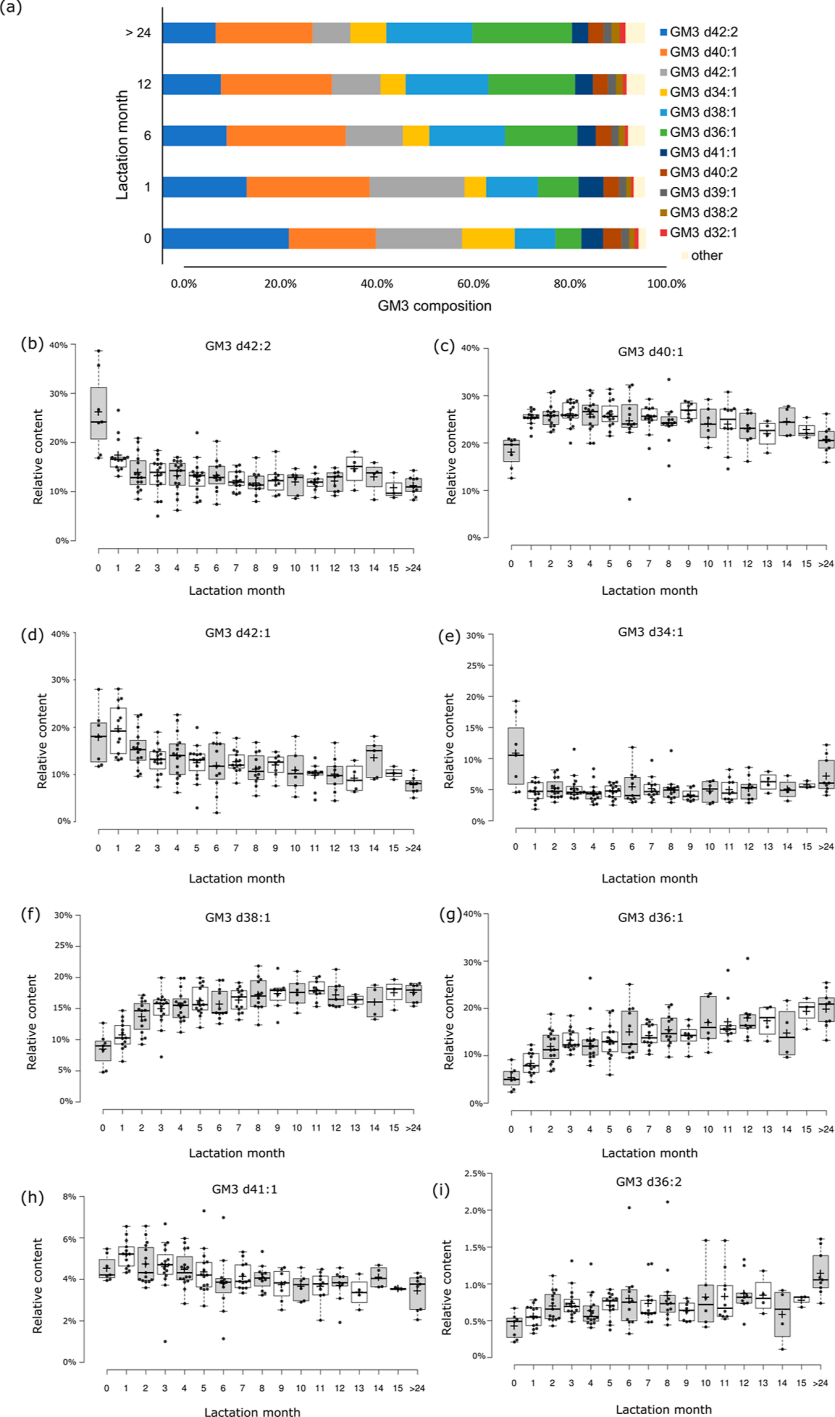
Compositional changes in GM3 profiles in human milk between different lactation stages. GM3 composition of HM samples collected in 5 points of lactation: 0 (colostrum), 1, 6, 12 and after 24 months of lactation presented as average % relative amount (a). Box-plots of relative content of GM3 d42:2 (b), GM3 d40:1 (c), GM3 d42:1 (d), GM3 d34:1 (e), GM3 d38:1 (f), GM3 d36:1 (g), GM3 d41:1 (h), and GM3 d36:2 (i) in HM samples collected in different lactation stages, starting from 0 to 15 month of lactation and after >24 month of lactation. Center lines show the medians; box limits indicate the 25th and 75th percentiles as determined by R software; whiskers extend 1.5 times the interquartile range from the 25th and 75th percentiles, outliers are represented by dots; crosses represent sample means; data points are plotted as open circles. *n* = 7, 13, 15, 16, 16, 15, 11, 13, 12, 8, 6, 10, 9, 4, 4, 3, and 9 sample points.

First, we evaluated the composition of GM3 profiles in HM samples with respect to the lactation period. Figure 2a shows the average composition of GM3 at four different lactation points: 0 month (colostrum) (*n* = 7), 1 month (*n* = 13), 6 month (*n* = 11), 12 month (*n* = 9), and >24 month (*n* = 9). The estimated relative content of GM3 species in all tested samples, as well as the average relative distribution in distinct months of lactation, are included in Tables S3 and S4, respectively.

As shown in Figure 2a, the most abundant GM3 through the course of lactation was GM3 d40:1, except for colostrum samples in which GM3 d42:2 was the most abundant one (average 26.2% ± SD 8.3%) and samples collected after 24 months of lactation with GM3 d36:1 being equally abundant as GM3 d40:1. In HM samples collected at various lactation periods, the average relative content of GM3 d40:1 varied from 18.3% (in colostrum samples) to 26.2% (3 months). The relative distribution of other GM3 species was more diverse during lactation. For instance, the relative content of GM3 d42:2—the most abundant GM3 in colostrum—decreased from 26.2% (average of colostrum samples, SD 8.3%) to 11% (SD 2.1%) in HM samples collected after 2 years of lactation. The trend of decreasing relative content was observed for GM3 species containing longer N-fatty acyl substituents: GM3 d42:2 (d18:1/24:1), GM3 d42:1 (d18:1/24:0), and GM3 d41:1 (d18:1/23:0). On the other hand, the relative content of GM3 d32:1, GM3 d36:1, GM3 d36:2, GM3 d38:1, and GM3 d38:2 containing 14:0, 18:0, 18:1, and 20:0 FA substituents increased in the later months of lactation.

Principal component analysis (PCA) was utilized to visualize the differences and similarities in GM3 profiles among the tested samples, which were grouped into four lactation stages: stage 1—0–6 months (*n* = 93), stage 2—7–12 months (*n* = 58), stage 3—13–19 months (*n* = 16), and stage 4—over 24 months (*n* = 9). Our results did not reveal any clustering based on the defined lactation stages (see Supporting Information, Figure S4a). However, colostrum samples were distinctly separated from samples collected during different lactation stages, although they exhibited significant interindividual variability. Comparing the HM samples collected in the earliest months of lactation with the HM samples collected after two years of lactation, a significant difference in GM3 composition is evident. According to the Mann–Whitney test, 9 out of 16 tested GM3 species content were different (*p* < 0.05, fold change (FC) > 50%) between the 1 month HM samples and >24 month HM samples, 6 increased and 3 decreased in HM samples collected after 24 month in comparison to 1 month HM samples (Table 2). A thorough examination of the monthly changes in the distribution of GM3 species provides further validation of the observed trends, as depicted in the box plots representing the two most significantly altered GM3 species: GM3 d42:1 [fold change (FC) = 2.28 for HM samples collected at 1 month compared with HM samples collected after 24 months) and GM3 d36:1 (FC = 0.42 for HM samples collected at 1 month compared with HM samples collected after 24 months).

**Table 2:** The List of Statistically Different GM3 Species between HM Samples Collected in 1 (*n* = 13) and after 24 month (*n* = 9) of Lactation

	fold change 1/24 month	*p* value
GM3 d36:1	0.42	0.000032
GM3 d38:1	0.61	0.000032
GM3 d42:1	2.28	0.000257
GM3 d36:2	0.48	0.000321
GM3 d38:2	0.62	0.000321
GM3 d42:2	1.56	0.000321
GM3 d41:1	1.52	0.000321
GM3 d32:1	0.51	0.003637
GM3 d36:0	0.54	0.013047

**Figure 3:**
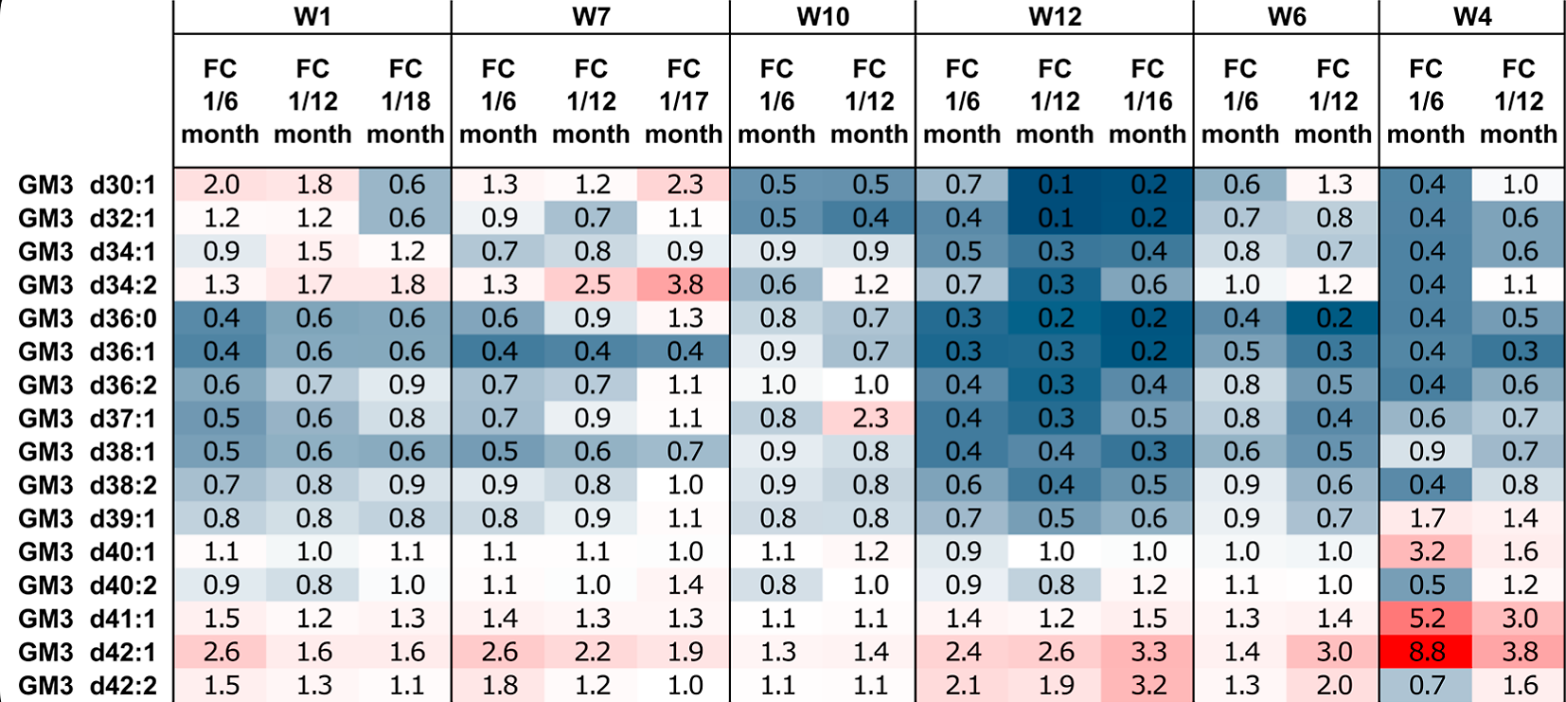
Changes in the relative amount of GM3 species in human milk (HM) samples collected from individual donors, comparing the 1 month samples to the later months of lactation (6, 12, 17, or 18). The fold change was calculated by dividing the relative amount of GM3 in the first month by the relative amount of GM3 in the later month. The color scheme indicates the observed trends: blue color represents a lower relative amount of GM3 in the first month compared to later months, red color indicates a higher relative amount in the first month of lactation compared to later months, and white color indicates no change between the tested samples (FC = 1). It is important to note that a single HM sample per month was used for the comparison.

Despite the lack of sample grouping based on donors, as demonstrated in the PCA score plot (refer to Figure S4b), the presence of interindividual variance is clearly evident in the box plots presented in Figure 2b–i. Figure 3 illustrates the changes in the relative abundance of GM3 species throughout lactation in human milk (HM) samples obtained from six different volunteers. We compared the GM3 profiles of HM samples collected at 6 month intervals, such as comparing the 1 month GM3 profile with the 6, 12, and 18 month profiles. While some general trends can be observed, it is important to note that the changes in GM3 profiles during different lactation stages are highly individualized for each donor. Furthermore, the alterations in the relative abundance of GM3 species can differ between the same lactation stages in HM samples collected by different volunteers. For instance, the content of GM3 d42:2 in the HM samples collected by woman 12 (W12) decreased by 3.2-fold from samples collected during her 1 month lactation to those collected during her 16 month lactation, whereas the relative amounts of GM3 in woman 7’s (W7) samples remained unchanged between her 1 and 17 month lactation periods.

## Discussion

In this study, we conducted a comprehensive analysis of the composition of GM3 molecular species in human milk throughout various lactation stages and across different donors. Our approach involved utilizing the RP-LC-Q-TOF-MS technique to provide detailed insights into the characteristics and dynamics of the ceramide component of gangliosides present in the human milk samples. Previous investigations concerning the composition of gangliosides in human milk have primarily focused on comparing the total content of specific ganglioside classes, such as GM3 and GD3, across different stages of lactation.^3,5,13,22^ Although certain studies have examined GM3 profiles in relation to the characteristics of the ceramide moiety, there is limited knowledge regarding the relative distribution of specific GM3 species in human milk throughout the lactation period.^13,15,16^

Previous research has demonstrated that the composition of gangliosides changes throughout the lactation period. These changes encompass the distribution of molecular species within specific ganglioside classes. However, it is important to note that the existing studies have primarily focused on human milk samples collected for up to 8 months of lactation. Further investigation is needed to explore the dynamics of ganglioside composition beyond this time point.^15,16^ In our study, we extended the analysis to human milk samples collected during advanced stages of lactation including samples obtained after 24 months of lactation. Our focus was on the GM3 fraction of gangliosides since the abundance of many GD3 species was very low in the majority of the samples, except from colostrum samples (the exemplary chromatogram is shown in the Figure S5). Therefore, we decided not to include them in the comparison. It was reported previously that the total content of GD3 decreases significantly in mature milk;^5,12^ hence, the determination of particular GD3 species was not possible because of their low abundance. However, it should be mentioned that the low abundance of GD3 can be connected with the lower binding affinity of GD3 than GM3 molecules to the SPE stationary phase used in this study (C-8 stationary phase).

By examining the GM3 fraction, we aimed to gain insights into the composition and dynamics of this specific ganglioside species throughout the extended lactation period. Human milk gangliosides exhibit a diverse array of ceramide moiety structures, encompassing variations in both the fatty acid composition linked to ceramide and the length and saturation of sphingosine. The significance of this structural diversification in human milk gangliosides remains poorly understood.^2^ Emerging data suggest that the specific characteristics of the ceramide component play a crucial role in antigenic properties and potentially contribute to immunosuppressive activity.^23−25^ As an example, it was reported that the ceramide structure is connected with the ability of lactosylceramides to be recognized by *Helicobacter pylori*.^23^ Gangliosides are highly abundant in the brain, where they constitute 10–12% of the lipid matter of the neuronal membrane.^26^ Mammalian brain gangliosides are primarily composed of sphingosine molecules with 18 and 20 carbon atoms and a C18 fatty acid amide, typically C18:0.^27,28^ This specific composition results in a relatively rigid structure and increased lateral self-association of gangliosides within the outer leaflet of the membrane.^28^ Ganglioside content and composition undergo significant changes as the brain develops and matures. During brain development, there are dynamic alterations in the levels and types of gangliosides present. Beginning with the simple GM3 and GD3 forms predominant in embryonic development, gangliosides become more complex as the brain matures into adulthood.^28,29^ Moreover, the ceramide-linked FA and sphingosine composition of brain tissue GA also changes with age, i.e., as shown by Rosenberg and Stern that the brain (rat and human) at birth contains almost exclusively C18 sphingosine, and later with organ maturation the content of C18 and C20 sphingosine was almost equal.^27^ Even though it has been demonstrated that the composition of HM gangliosides varies with lactation stage, a connection between the changes in GA composition in HM and the brain remains unknown and should be investigated in much more detail. The reason for its relevance is the similar composition of FAs in the HM and brain gangliosides. FAs that predominate in HM gangliosides are also the most abundant in the brain at the early stage of development.^2^

In our study, we employed the MS/MS technique to elucidate the sphingosine and N-fatty acid compositions of the identified GM3 species. The GM3 species detected in this study were found to be consistent with those reported in previous studies.^13,16,30^ An interesting finding in our study was the detection of multiple possible ceramide structures for GM3 species with the same molecular mass, which was achieved through the interpretation of the MS/MS spectra. For instance, the GM3 d40:1 species was found to contain C18:1 sphingosine and 22:0 N-fatty acid, but we also observed the presence of the ions corresponding to the d16:1/24:0 structure. However, it is worth noting that these isomers could not be separated by chromatography method employed in our study, and the possible structures are proposed only based on the observed fragment ions of LCB. The coexistence of multiple ceramide structures within the same GM3 species highlights the complexity and diversity of ganglioside composition and underscores the challenges in fully resolving and characterizing these isomers.

We observed that the greatest difference in the ceramide moiety of GM3 occurs between samples collected at the earliest stage of lactation and samples collected later (after 2 years of lactation). The most abundant and constant relative amount through the course of lactation was GM3 d40:1, except for colostrum samples in which GM3 d42:2 was the most abundant one and samples collected after 24 months with slightly higher average content of GM3 d36:1 (20.6% for GM3d36:1 and 20.1% for GM3 d40:1). This is in accordance with the study of Ma et al.,^16^ where the most dominant molecular species throughout the lactation period were GM3 d40:1 and GM3 d42:1. However, in our study, the second most abundant GM3 were GM3 d42:1 during the first 2 months of lactation, GM3 d38:1 between 3 and 11 months, and GM3 d36:1 after 12 months of lactation.

The relative content of GM3 containing very long N-fatty acyl (N-FA) substituents with >22 carbon atoms such as GM3 d42:2 (d18:1/24:1), GM3 d42:1 (d18:1/24:0), and GM3 d41:1 (d18:1/23:0) decreased in the course of lactation. On the opposite, the content of GM3 species containing 14:0, 18:0, 18:1, and 20:0 N-FA substituents such as GM3 d32:1, GM3 d36:1, GM3 d36:2, and GM3 d38:1 increased in the later months of lactation. A different pattern was observed for colostrum samples, where the relative content of GM3 d40:1 and GM3 d42:1 was lower in comparison to that in the sample collected at 1 month of lactation, and relative content of GM3 d34:1 was higher than that in samples collected at later points of lactation. A similar pattern in the changes of the relative distribution of the GM3 molecular species was observed by Ma et al. for HM samples collected between 1 and 8 months of lactation.^16^ Martin-Sosa and coauthors^15^ also tested the differences in ceramide moiety structure of GA between HM samples collected at different lactation stages. They observed that FAs from C10 to C15 were at very low level in colostrum samples and their content increased in later lactation stages, whereas LCFA (FA from C16 to C21) content diminished after colostrum, and very long-chain fatty acid (VLCFA) content was constant among tested samples.^15^

In addition to the general trends observed in the dynamics of GM3 during lactation, we also observed individual differences among the donors. It was notable that the magnitude of the decrease or increase in specific GM3 species was not consistent among donors when comparing the same lactation periods (e.g., 1 month vs 6 months), suggesting that the GM3 composition is highly specific for individual mothers. This variability in GM3 dynamics can be attributed to various factors, including maternal diet, which is well-documented to influence the composition of human milk. Specifically, the fatty acid composition of human milk is known to be influenced by maternal dietary factors.^31−33^ However, the relationship between gangliosides and factors that could potentially influence their composition still requires further investigation. A study conducted by Tan and colleagues aimed to address this by examining and comparing the distribution of gangliosides in human milk from Chinese mothers across different characteristic groups.^4^ The results of their study revealed that there were no significant differences in ganglioside content based on factors such as the city of residence, infant gender, delivery mode, pregnancy age, and blood group. There was no significant difference between the groups at different time points. However, the study measured only the total content of different ganglioside fractions up to 6 months. Therefore, follow-up studies, including samples collected at prolonged lactations and regarding the molecular distribution of GM3 species, are required to explore this topic.

The interindividual variability observed in this study poses challenges for statistical analysis and classification of samples according to lactation month/period. Additionally, literature reports suggest that the composition of human milk, including the GM3 profile, may be influenced by circadian variations. Despite our efforts to standardize sample collection time (evening, same day of the month), it is possible that differences observed in the GM3 profiles among different volunteers could be attributed to variations in the timing of human milk collection. Furthermore, the unequal sample size and limited number of samples in certain lactation months (e.g., month 15 with only 3 human milk samples) can be considered as limitations of the study. However, it is important to highlight that this study still contributes new information and valuable insights into the dynamics of GM3 profiles across lactation. Certainly, given the potential impact of the ceramide structure in GM3 species on newborns’ defense against infection and brain development, further research should be conducted to explore the compositional and functional aspects of GM3 species in human milk. While this study identified differences in the molecular distribution of GM3 based on lactation duration and individual donors, their significance for infant health and development needs to be further investigated and defined. It is crucial to evaluate whether the observed changes in the GM3 composition are sufficient to elicit a biological effect. This becomes particularly important when considering the optimization of infant formula composition to closely match the GM3 profile found in human milk.

## Data Availability

The data that support the findings of this study are available from the corresponding author upon request.

## Supporting Information Available

Inclusion criteria for HM sample donors; characteristics of the samples used in the study and applied grouping of the samples; results of the evaluation of the precision of the extraction procedure for the GM3 included in the comparative analysis; MS/MS spectrum of GM3 d40:1; MS/MS spectrum of GM3 d42:3; exemplary extracted ion chromatograms of GM3 d41:2 and GM3 d43:2 analyzed in the HM sample; percent relative amount of GM3 species in tested HM samples; average percent relative distribution of GM3 species throughout the lactation; and PCA score plot of GM3 profiles of human milk samples collected at different lactation points (PDF)

## Abbreviations

GA: ganglioside
GC: gas chromatography
HM: human milk
LC-HRMS: liquid chromatography coupled to high-resolution mass spectrometry
LC-MS: liquid chromatography coupled to mass spectrometry
LCB: long-chain base
LCFA: long-chain fatty acid
MeOH: methanol
MFGM: milk fat globule membrane
N-FA: N-fatty acyl
SA: sialic acid
VLCFA: very long-chain fatty acid
