# Supplementary material for: Characterization of GM3 Gangliosides in Human Milk throughout Lactation: Insights from the Analysis with the Use of Reversed-Phase Liquid Chromatography Coupled to Quadrupole Time-Of-Flight Mass Spectrometry
Source: J Agric Food Chem. 2023 Nov 13;71(46):17899–908. doi: 10.1021/acs.jafc.3c04489 (PMC10682988; doi:10.1021/acs.jafc.3c04489)
Supplement: Supplementary file 1 — jf3c04489_si_001.pdf [file jf3c04489_si_001.pdf]

SUPPORTING INFORMATION

Characterization of GM3 gangliosides in human milk throughout lactation: insights from the analysis with the use of reversed-phase liquid chromatography coupled to quadrupole time-of-flight mass spectrometry

Weronika Hewelt-Belka[1]*, Michał Młynarczyk[1], Dorota Garwolińska[1], Agata Kot-Wasik[1]

[1] Department of Analytical Chemistry, Faculty of Chemistry, Gdańsk University of Technology, 80-233 Gdańsk, Poland

* weronika.belka@pg.edu.pl

Table of contents:

Inclusion criteria for HM sample donors.	p. 3
Table S1. Characteristics of the samples used in the study and applied grouping of the samples.	p. 4
Figure S1. The MS/MS spectrum of GM3 d40:1 acquired in positive ionization mode.	p. 8
Figure S2. The MS/MS spectrum of GM3 d42:3 acquired in positive ionization mode.	p. 8
Figure S3. The exemplary extracted ion chromatograms of GM3 d41:2 and GM3 d43:2 analysed in HM sample with the use of RP-LC-Q-TOF-MS technique in negative ionisation mode.	p. 8
Table S2. Results of the evaluation of the precision of the extraction procedure for the GM3 included in the comparative analysis	p. 9
Table S3. The percent relative amount of GM3 species in tested HM samples.	p. 10
Table S4. Average percent relative distribution of GM3 species throughout the lactation.	p. 18
Figure S4. PCA score plot of GM3 profiles of human milk samples collected at different lactation point.	p. 20
Figure S5. Exemplary extracted ion chromatograms of the GD3 species analyzed in colostrum sample with the use of RP-LC-Q-TOF-MS technique in negative ionization mode.	p. 20

Inclusion criteria for HM sample donors:

Women and their children participating in the study had to meet the inclusion criteria:

- mothers of singleton infants breastfeeding exclusively/fully lactating for the first 6 months,
- breastfeeding mothers of singleton infants at age 6 months or higher (complementary foods starting at 6 months was permitted for all infants as per American Academy of Pediatrics (AAP) guidelines and World Health Organization recommendations as long as their sole milk source was maternal breast milk up to the age of 1 year,
- infants had to be ≥35 weeks' gestation and in good general health at the time of enrollment.

Subject exclusion criteria included:

- mothers diagnose: preexisting type I or II diabetes, hypertension, parathyroid disease, and uncontrolled thyroid disease.
- twins or multiple births
- infants < 35 weeks' gestation; with a history of 72 hours in the NICU; any inborn error of metabolism; the history of congenital anomalies; or a history of consuming 10 of their diet as formula at the time of enrollment
- combined feeding at the time of enrollment (i.e., partially breastfeeding and formula-feeding their infants).

Table S1. Characteristics of the samples used in the study and applied grouping of the samples. The sample number pattern is WX_Yn, where 'W' stands for the woman, 'X' is the woman's number, 'Y' denotes the month of lactation, and 'n' differentiate separates samples collected in the same lactation month.

Sample name	Lactation month	Woman
**W1_1**	1	1
**W1_2**	2	1
**W1_3**	3	1
**W1_4**	4	1
**W1_5**	5	1
**W1_6**	6	1
**W1_7**	7	1
**W1_8**	8	1
**W1_9**	9	1
**W1_10**	10	1
**W1_11**	11	1
**W1_12**	12	1
**W1_13**	13	1
**W1_14**	14	1
**W1_15**	15	1
**W1_16**	16	1
**W1_18**	18	1
**W1_19**	19	1
**W2_3**	3	2
**W2_4**	4	2
**W2_5**	5	2
**W2_6**	6	2
**W2_7**	7	2
**W2_8**	8	2
**W2_9**	9	2
**W2_11**	11	2
**W2_12**	12	2
**W3_1**	1	3
**W3_2**	2	3
**W3_3**	3	3
**W3_4**	4	3
**W3_5**	5	3
**W3_6**	6	3
**W3_7**	7	3
**W4_1**	1	4
**W4_2**	2	4
**W4_3**	3	4
**W4_4**	4	4
**W4_5**	5	4
**W4_6**	6	4

**W4_7**	7	4
**W4_8**	8	4
**W4_9**	9	4
**W4_11**	11	4
**W4_12**	12	4
**W5_1**	1	5
**W5_2**	2	5
**W5_3**	3	5
**W5_4**	4	5
**W5_5**	5	5
**W5_6**	6	5
**W5_7**	7	5
**W5_8**	8	5
**W5_9**	9	5
**W6_1**	1	6
**W6_2**	2	6
**W6_4**	4	6
**W6_5**	5	6
**W6_6**	6	6
**W6_7**	7	6
**W6_9**	9	6
**W6_10**	10	6
**W6_11**	11	6
**W6_12**	12	6
**W7_1**	1	7
**W7_2**	2	7
**W7_3**	3	7
**W7_4**	4	7
**W7_5**	5	7
**W7_6**	6	7
**W7_7**	7	7
**W7_8**	8	7
**W7_9**	9	7
**W7_10**	10	7
**W7_11**	11	7
**W7_12**	12	7
**W7_13**	13	7
**W7_14**	14	7
**W7_15**	15	7
**W7_17**	17	7
**W8_1**	1	8
**W8_2**	2	8
**W8_3**	3	8
**W8_4**	4	8
**W8_5**	5	8

**W9_1**	1	9
**W9_2**	2	9
**W9_3**	3	9
**W10_1**	1	10
**W10_2**	2	10
**W10_3**	3	10
**W10_4**	4	10
**W10_5**	5	10
**W10_6**	6	10
**W10_7**	7	10
**W10_8**	8	10
**W10_9**	9	10
**W10_10**	10	10
**W10_11**	11	10
**W10_12**	12	10
**W10_13**	13	10
**W10_14**	14	10
**W11_2**	2	11
**W11_3**	3	11
**W11_4**	4	11
**W11_5**	5	11
**W12_1**	1	12
**W12_2**	2	12
**W12_3**	3	12
**W12_4**	4	12
**W12_5**	5	12
**W12_6**	6	12
**W12_7**	7	12
**W12_8**	8	12
**W12_9**	9	12
**W12_11a**	11	12
**W12_11b**	11	12
**W12_12**	12	12
**W12_13**	13	12
**W12_14**	14	12
**W12_15**	15	12
**W12_16**	16	12
**W13_8**	8	13
**W13_10**	10	13
**W13_11**	11	13
**W13_12**	12	13
**W14_2**	2	14
**W14_3**	3	14
**W14_4**	4	14

**W14_5**	5	14
**W14_6**	6	14
**W14_7**	7	14
**W14_8**	8	14
**W14_10**	10	14
**W14_11**	11	14
**W14_12**	12	14
**W15_1**	1	15
**W15_2**	2	15
**W15_3**	3	15
**W15_4**	4	15
**W15_5**	5	15
**W15_6**	6	15
**W15_7a**	7	15
**W15_7b**	7	15
**W15_8a**	8	15
**W15_8b**	8	15
**W16_1**	1	16
**W16_2**	2	16
**W16_3**	3	16
**W16_4**	4	16
**W16_5**	5	16
**W16_7**	7	16
**W16_8**	8	16
**W17_0**	0	17
**W17_1**	1	17
**W17_2**	2	17
**W17_3a**	3	17
**W17_3b**	3	17
**W17_4a**	4	17
**W17_4b**	4	17
**W17_5**	4	17
**W18_36**	36	18
**W19_35**	35	19
**W20_29**	29	20
**W20_30**	30	20
**W21_29**	29	21
**W22_28**	28	22
**W22_29**	29	22
**W22_30**	30	22
**W23_26**	26	23
**W24_0**	0	24
**W25_0**	0	25
**W26_0**	0	26
**W27_0**	0	27

**W28_0**	0
**W29_0**	0

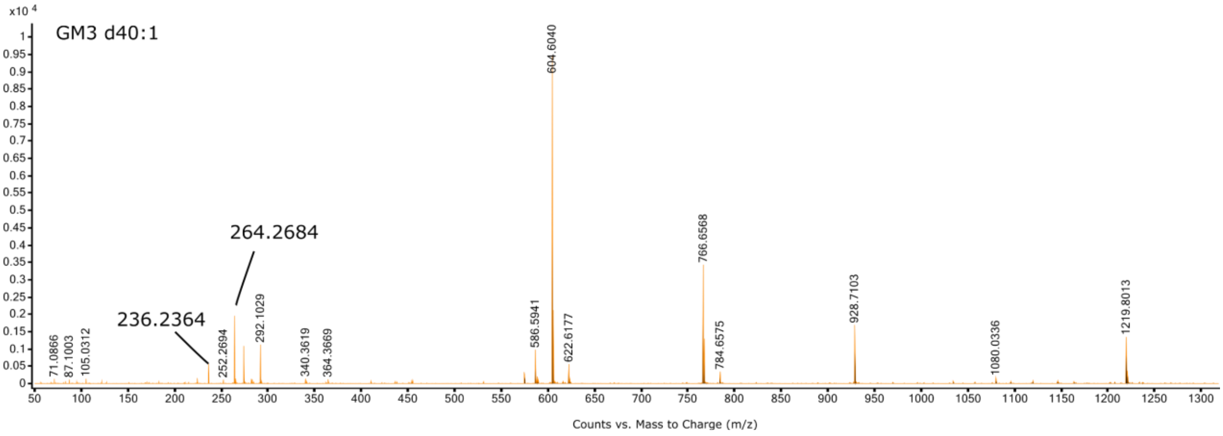

Figure S1. The MS/MS spectrum of GM3 d40:1 acquired in positive ionization mode.

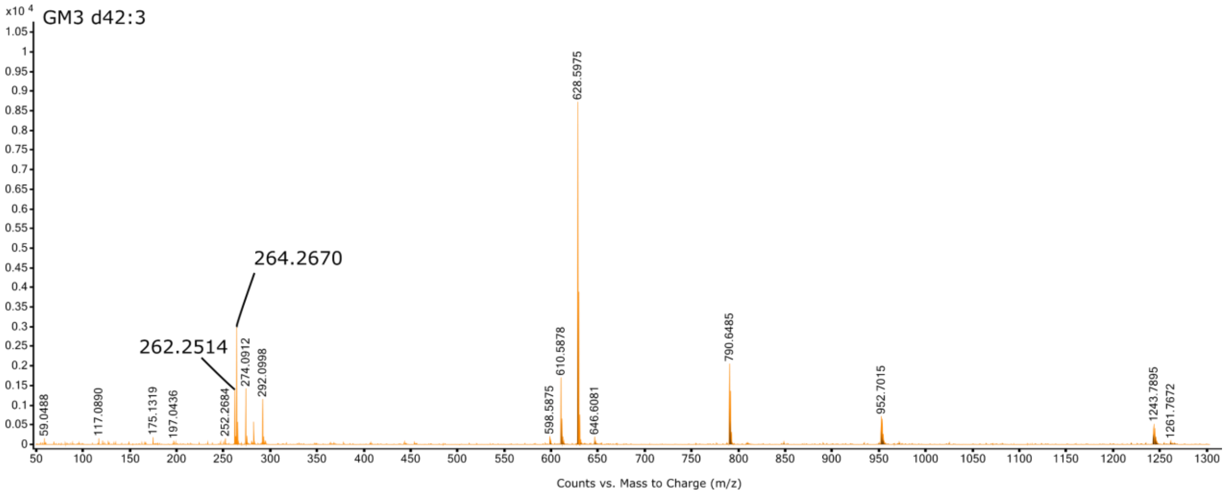

Figure S2. The MS/MS spectrum of GM3 d42:3 acquired in positive ionization mode.

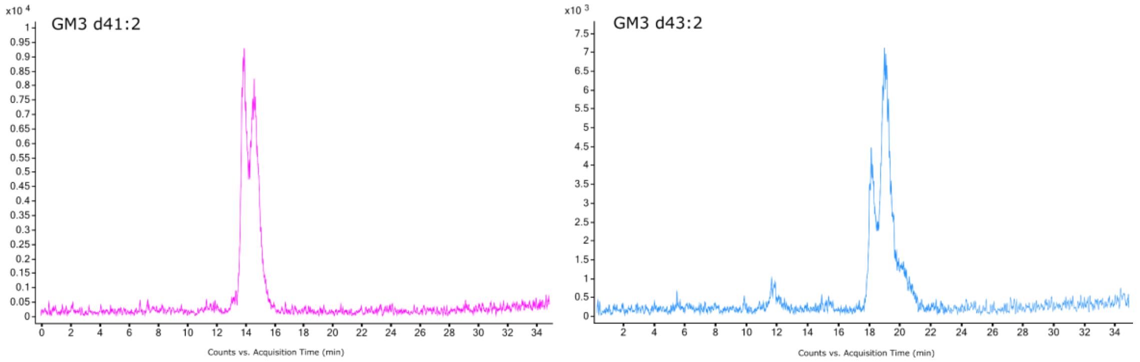

Figure S3. The exemplary extracted ion chromatograms of GM3 d41:2 and GM3 d43:2 analysed in HM sample with the use of RP-LC-Q-TOF-MS technique in negative ionisation mode.

Table S2. Results of the evaluation of the precision of the extraction procedure for the GM3 included in the comparative analysis.

	%RSD of normalized peak area 12.5 ug/mL (n=3)	%RSD of normalized peak area 2.5 ug/mL (n=3)	%RSD of normalized peak area Human milk pooled sample (n=5)
GM3 d30:1	19	22	20
GM3 d32:1	12	29	12
GM3 d34:1	8	24	18
GM3 d34:2	15	24	14
GM3 d36:0	3	54	25
GM3 d36:1	13	6	19
GM3 d36:2	10	12	15
GM3 d37:1	15	14	15
GM3 d38:1	5	7	8
GM3 d38:2	9	15	18
GM3 d39:1	1	6	4
GM3 d40:1	2	2	6
GM3 d40:2	1	4	9
GM3 d41:1	5	5	14
GM3 d42:1	1	20	22
GM3 d42:2	3	10	14

Table S3. The percent relative amount of GM3 species in tested HM samples.

Sample name	Lactatio n month	Wo man	Lactation period	GM3 d39:1	GM3 d38:2	GM3 d40:2	GM3 d41:1	GM3 d36:2	GM3 d36:0	GM3 d37:1	GM3 d42:2	GM3 30:1	GM3 d32:1	GM3 d40:1	GM3 d38:1	GM3 d42:1	GM3 d36:1	GM3 d34:1	GM3 34:2
W1_1	1	1	1	1.13	0.88	2.55	4.52	0.55	0.74	0.58	16.79	0.17	0.87	25.98	9.30	21.42	8.04	6.13	0.33
W1_2	2	1	1	1.37	1.10	2.85	3.88	0.84	1.14	0.90	13.52	0.14	1.00	23.55	13.16	14.70	13.59	7.83	0.43
W1_3	3	1	1	1.25	0.94	2.29	4.87	0.70	0.86	0.79	17.63	0.14	0.87	25.00	7.24	18.97	11.81	6.36	0.27
W1_4	4	1	1	1.40	1.14	3.14	4.12	0.61	1.13	0.89	14.27	0.08	0.52	28.13	14.36	13.45	12.01	4.54	0.20
W1_5	5	1	1	1.33	1.23	3.17	3.71	0.78	1.15	0.97	13.41	0.09	0.65	26.66	14.64	13.12	13.81	5.05	0.22
W1_6	6	1	1	1.45	1.28	2.85	3.10	0.96	1.72	1.17	10.86	0.09	0.74	23.00	17.49	8.26	19.97	6.83	0.26
W1_7	7	1	2	1.50	1.07	3.43	4.22	0.60	1.05	0.88	14.09	0.12	0.74	24.84	14.30	17.70	11.05	4.22	0.19
W1_8	8	1	2	1.22	0.99	2.98	4.36	0.65	1.04	0.87	16.91	0.17	0.88	23.84	12.37	16.79	12.10	4.64	0.20
W1_9	9	1	2	1.43	0.97	3.40	4.57	0.49	0.85	0.78	18.16	0.08	0.51	27.85	12.75	14.80	9.84	3.37	0.14
W1_10	10	1	2	1.48	1.07	2.96	4.03	0.63	1.34	1.00	13.25	0.14	0.57	25.27	15.90	13.98	13.66	4.58	0.15
W1_11	11	1	2	1.47	1.01	2.97	3.23	0.93	1.18	0.98	12.35	0.26	1.31	23.03	19.31	9.91	15.63	6.18	0.27
W1_12	12	1	2	1.34	1.15	3.04	3.79	0.73	1.33	0.93	13.01	0.09	0.72	27.02	15.28	13.26	13.96	4.17	0.19
W1_13	13	1	3	1.44	1.29	4.00	2.53	0.86	1.36	0.96	18.11	0.13	1.07	23.60	16.19	6.36	16.08	5.77	0.24
W1_14	14	1	3	1.39	0.90	2.61	4.69	0.46	1.01	0.76	15.91	0.12	0.70	27.10	13.28	18.09	9.68	3.18	0.14
W1_15	15	1	3	1.30	1.06	2.45	3.53	0.68	1.65	0.97	13.87	0.19	1.25	25.39	14.75	11.74	15.61	5.34	0.22
W1_16	16	1	3	1.42	0.88	2.61	4.56	0.42	0.59	0.61	16.15	0.12	0.65	26.65	13.91	18.23	10.06	3.04	0.10
W1_18	18	1	3	1.44	1.03	2.60	3.38	0.61	1.30	0.70	14.95	0.27	1.35	23.96	15.90	13.17	13.97	5.20	0.19
W1_19	19	1	3	1.36	0.89	2.72	4.13	0.43	1.00	0.40	18.63	0.08	0.65	25.71	14.38	15.35	11.00	3.17	0.08
W2_3	3	2	1	1.75	1.24	3.64	4.74	0.61	1.08	1.01	16.58	0.05	0.32	27.54	14.65	12.71	10.21	3.69	0.18
W2_4	4	2	1	1.95	1.19	2.91	4.58	0.53	1.01	0.95	11.01	0.09	0.38	30.59	15.76	14.14	10.59	4.16	0.17

W2_5	5	2	1	1.91	1.12	2.93	4.84	0.48	0.81	0.88	11.72	0.06	0.31	31.38	15.50	14.32	10.08	3.49	0.15
W2_6	6	2	1	1.89	1.11	2.79	4.91	0.52	1.07	0.89	10.03	0.06	0.34	32.27	14.96	14.94	10.50	3.58	0.14
W2_7	7	2	2	2.13	1.29	2.99	4.70	0.61	1.36	1.09	11.73	0.09	0.42	27.21	18.13	11.38	12.86	3.85	0.16
W2_8	8	2	2	1.59	1.01	2.94	4.03	0.44	0.97	0.78	10.56	0.08	0.40	33.42	15.40	14.97	9.73	3.54	0.15
W2_9	9	2	2	2.06	1.55	3.14	3.46	0.80	1.39	1.40	9.36	0.06	0.39	25.34	21.48	7.61	17.63	4.17	0.15
W2_11	11	2	2	1.84	1.31	3.05	3.73	0.56	1.24	0.99	8.79	0.07	0.35	30.78	20.16	10.42	13.06	3.51	0.13
W2_12	12	2	2	1.91	1.53	3.16	3.08	0.84	1.65	1.31	9.95	0.16	0.75	23.11	21.31	6.28	18.90	5.84	0.23
W3_1	1	3	1	1.29	0.79	3.00	5.57	0.43	0.40	0.51	16.41	0.12	0.46	27.12	8.59	26.07	5.88	3.24	0.14
W3_2	2	3	1	1.59	1.10	3.10	5.02	0.56	0.52	0.84	11.97	0.04	0.27	30.63	14.72	15.64	10.03	3.84	0.13
W3_3	3	3	1	1.96	1.14	3.31	6.68	0.63	0.68	0.94	7.98	0.05	0.36	29.20	16.41	14.73	11.44	4.35	0.14
W3_4	4	3	1	1.07	0.78	2.86	4.70	0.40	0.56	0.57	15.33	0.07	0.41	26.88	11.20	22.64	7.94	4.45	0.13
W3_5	5	3	1	1.48	1.17	2.75	3.53	0.78	1.34	1.00	7.80	0.13	0.69	25.13	19.94	11.78	16.28	5.99	0.21
W3_6	6	3	1	1.24	0.83	3.03	3.99	0.48	0.77	0.63	13.31	0.06	0.41	31.84	12.56	16.50	10.80	3.43	0.10
W3_7	7	3	2	1.34	1.05	2.89	3.34	0.64	0.92	0.86	9.80	0.06	0.59	27.38	17.64	10.84	16.55	5.98	0.13
W4_1	1	4	1	1.92	1.17	3.09	5.87	0.78	0.78	0.96	14.39	0.10	0.50	25.84	12.29	16.87	10.44	4.73	0.28
W4_2	2	4	1	1.67	1.35	2.70	3.60	1.11	1.63	1.24	9.90	0.13	0.82	22.30	16.12	10.12	18.81	8.17	0.34
W4_3	3	4	1	1.59	1.14	3.48	4.53	0.69	1.01	0.85	15.72	0.09	0.46	28.50	13.16	12.35	12.08	4.17	0.17
W4_4	4	4	1	1.56	1.24	3.65	3.53	0.64	1.36	0.89	16.99	0.07	0.39	25.51	17.01	9.19	13.15	4.61	0.21
W4_5	5	4	1	1.71	1.64	4.84	2.72	0.77	1.53	1.02	21.98	0.09	0.46	21.50	18.24	2.95	15.00	5.30	0.23
W4_6	6	4	1	1.13	2.70	5.86	1.14	2.03	1.96	1.50	20.26	0.26	1.22	8.12	14.31	1.92	25.08	11.80	0.71
W4_7	7	4	2	1.95	1.23	2.84	4.74	0.48	0.62	0.88	13.06	0.17	0.61	25.11	15.32	14.17	12.76	5.82	0.26
W4_8	8	4	2	1.84	1.28	2.87	3.33	0.77	1.31	1.04	11.27	0.10	0.58	24.11	19.30	8.63	18.20	5.23	0.16

W4_9	9	4	2	1.77	1.12	2.67	3.81	0.72	1.17	0.99	11.32	0.14	0.66	24.93	17.91	10.88	16.34	5.35	0.22
W4_11	11	4	2	1.41	1.53	2.91	2.03	1.23	1.66	1.27	10.39	0.09	0.74	16.96	19.86	4.63	28.05	7.03	0.21
W4_12	12	4	2	1.33	1.40	2.47	1.93	1.33	1.50	1.31	9.19	0.11	0.90	16.12	18.56	4.46	30.56	8.57	0.26
W5_1	1	5	1	1.89	1.39	3.65	4.42	0.54	0.32	0.62	16.60	0.22	0.80	25.29	14.68	13.61	10.51	5.29	0.17
W5_2	2	5	1	1.80	1.16	2.97	4.00	0.43	0.56	0.62	11.96	0.12	0.73	26.64	17.17	13.59	13.51	4.73	0.01
W5_3	3	5	1	2.58	1.30	3.39	5.78	0.58	0.51	0.07	14.36	0.13	0.67	25.86	15.85	13.22	11.23	4.36	0.11
W5_4	4	5	1	1.71	1.46	3.60	4.03	0.85	1.20	1.04	15.37	0.17	0.69	24.05	16.07	11.65	12.45	5.43	0.23
W5_5	5	5	1	2.00	1.55	3.77	3.51	0.87	1.67	1.20	14.92	0.15	0.71	22.29	18.64	7.89	15.06	5.52	0.23
W5_6	6	5	1	1.41	1.45	3.35	2.47	0.90	2.10	1.06	12.48	0.18	1.01	22.25	19.56	5.24	18.99	7.35	0.21
W5_7	7	5	2	1.43	1.08	2.65	3.58	0.60	1.71	0.91	12.27	0.09	0.46	25.79	17.87	11.80	14.93	4.69	0.14
W5_8	8	5	2	1.82	1.40	3.01	3.24	0.74	1.35	1.12	10.61	0.08	0.46	24.95	21.85	7.58	17.28	4.39	0.11
W5_9	9	5	2	1.51	1.07	3.15	2.53	0.49	1.19	0.74	13.67	0.10	0.55	28.59	17.96	12.45	12.54	3.38	0.09
W6_1	1	6	1	1.17	0.80	3.06	5.19	0.39	0.56	0.54	20.60	0.07	0.30	25.18	8.90	24.08	6.50	2.54	0.12
W6_2	2	6	1	1.30	1.18	3.59	3.89	0.66	0.43	0.64	20.11	0.16	0.72	23.62	12.21	15.72	11.17	4.42	0.18
W6_4	4	6	1	1.34	0.95	2.94	4.60	0.47	1.13	0.68	16.17	0.08	0.36	27.30	13.04	19.24	8.87	2.70	0.14
W6_5	5	6	1	1.57	1.06	2.96	4.21	0.64	1.58	0.90	13.74	0.13	0.52	25.62	15.94	14.37	12.68	3.92	0.18
W6_6	6	6	1	1.26	0.88	2.78	3.91	0.48	1.25	0.72	16.26	0.11	0.42	26.01	14.24	16.63	11.84	3.08	0.12
W6_7	7	6	2	1.52	1.08	2.97	3.85	0.58	1.33	0.91	14.02	0.12	0.53	25.65	17.21	12.91	13.78	3.41	0.13
W6_9	9	6	2	1.47	1.00	2.67	2.95	0.51	1.46	0.86	11.65	0.10	0.49	28.84	18.30	12.82	13.65	3.11	0.13
W6_10	10	6	2	1.39	0.98	2.99	3.94	0.48	1.41	0.87	12.57	0.06	0.38	29.21	17.21	10.91	14.82	2.70	0.08
W6_11	11	6	2	1.28	0.97	2.90	3.52	0.52	1.60	0.81	13.72	0.08	0.44	26.91	17.36	10.74	16.09	2.96	0.09

W6_12	12	6	2	1.67	1.26	2.95	3.68	0.77	2.69	1.24	10.06	0.05	0.40	26.31	18.67	8.03	18.66	3.46	0.10
W7_1	1	7	1	1.41	0.93	2.71	5.39	0.65	0.84	0.87	13.12	0.20	0.61	25.36	10.20	25.77	7.26	4.34	0.34
W7_2	2	7	1	1.81	1.19	2.72	4.74	0.92	1.17	1.13	8.46	0.14	0.60	25.55	16.70	13.46	15.65	5.54	0.23
W7_3	3	7	1	1.69	1.23	3.22	4.18	0.73	1.27	1.04	12.09	0.11	0.50	25.55	15.94	14.03	13.34	4.89	0.18
W7_4	4	7	1	1.81	1.15	2.45	3.93	0.52	1.54	1.24	8.36	0.10	0.50	23.52	19.89	10.35	19.99	4.42	0.23
W7_5	5	7	1	1.73	1.21	2.56	3.57	0.86	1.47	1.24	8.10	0.13	0.65	23.71	19.35	9.89	19.12	6.21	0.22
W7_6	6	7	1	1.75	1.05	2.40	3.79	0.95	1.50	1.28	7.43	0.15	0.65	23.63	19.56	9.81	19.85	5.96	0.26
W7_7	7	7	2	1.66	1.20	2.74	3.88	0.78	1.27	1.18	9.75	0.09	0.45	25.66	18.55	11.99	16.27	4.37	0.15
W7_8	8	7	2	1.81	1.19	2.52	3.99	0.59	1.50	1.38	8.00	0.11	0.56	24.31	20.14	10.05	20.79	2.93	0.15
W7_9	9	7	2	1.66	1.02	2.57	4.28	0.62	1.22	1.02	9.10	0.10	0.44	28.26	18.01	13.49	14.04	4.02	0.16
W7_10	10	7	2	1.40	1.12	1.93	3.56	0.80	1.60	1.39	8.62	0.22	0.85	22.66	17.98	9.47	22.54	5.63	0.22
W7_11	11	7	2	1.63	1.09	2.92	4.16	0.71	0.87	0.88	11.07	0.08	0.57	27.26	17.71	10.24	16.38	4.33	0.10
W7_12	12	7	2	1.55	1.15	2.73	4.14	0.88	0.96	0.93	11.18	0.17	0.88	23.72	16.48	11.72	18.06	5.32	0.14
W7_13	13	7	3	1.49	1.17	2.34	3.25	0.74	0.75	0.69	10.26	0.27	1.31	21.69	17.26	10.57	20.03	7.91	0.28
W7_14	14	7	3	1.71	1.14	2.18	3.66	0.91	1.28	1.13	8.35	0.17	1.08	21.54	18.78	9.03	21.64	7.25	0.15
W7_15	15	7	3	1.64	1.22	2.30	3.53	0.82	1.83	1.07	9.67	0.14	0.83	21.12	18.15	8.96	22.15	6.43	0.14
W7_17	17	7	3	1.25	0.92	2.00	4.20	0.58	0.67	0.78	13.01	0.08	0.57	25.55	15.27	13.85	16.45	4.72	0.09
W8_1	1	8	1	1.78	1.29	4.48	4.65	0.68	0.77	0.84	22.00	0.13	0.61	24.14	11.07	13.08	7.90	6.20	0.38
W8_2	2	8	1	1.33	0.80	2.61	6.16	0.52	0.68	0.67	12.83	0.08	0.41	30.91	9.24	22.64	7.17	3.77	0.19
W8_3	3	8	1	1.45	1.05	3.17	5.43	0.63	0.92	0.81	15.55	0.06	0.33	28.87	11.46	16.64	9.82	3.66	0.16
W8_4	4	8	1	1.79	1.04	2.93	5.77	0.57	0.99	0.86	11.72	0.06	0.30	30.02	13.64	16.74	9.84	3.58	0.16
W8_5	5	8	1	1.58	1.22	2.91	4.44	0.69	1.16	1.02	10.50	0.07	0.43	29.19	16.41	13.12	12.53	4.53	0.20

W9_1	1	9	1	1.58	1.07	3.32	4.94	0.68	0.68	0.82	15.01	0.08	0.38	27.49	11.60	17.53	9.80	4.78	0.24
W9_2	2	9	1	1.79	1.26	3.18	4.33	0.88	0.91	1.07	13.61	0.11	0.50	24.83	15.48	12.61	14.02	5.20	0.24
W9_3	3	9	1	1.48	1.08	3.04	4.42	0.64	1.09	0.93	14.87	0.07	0.39	25.86	14.13	14.98	12.25	4.56	0.20
W10_1	1	10	1	1.66	1.18	3.31	4.33	0.75	1.04	0.99	16.37	0.15	0.58	25.31	13.01	13.38	11.30	6.29	0.33
W10_2	2	10	1	1.92	1.49	3.47	3.99	0.79	1.47	1.21	12.86	0.15	0.62	24.24	16.19	9.63	14.68	6.96	0.32
W10_3	3	10	1	2.01	1.51	3.33	4.31	1.01	1.49	1.36	11.62	0.17	0.80	23.49	16.38	8.83	16.11	7.28	0.29
W10_4	4	10	1	2.08	1.40	3.50	4.12	0.89	1.43	1.30	11.63	0.45	1.44	22.99	15.79	8.13	15.73	8.35	0.76
W10_5	5	10	1	1.86	1.14	3.11	5.13	0.70	1.05	0.99	13.00	0.44	1.26	26.32	13.81	13.42	11.19	6.19	0.40
W10_6	6	10	1	2.02	1.36	3.92	4.09	0.77	1.31	1.29	15.05	0.32	1.27	23.96	14.34	10.21	12.45	7.12	0.51
W10_7	7	10	2	1.83	1.10	3.29	5.13	0.60	0.80	0.97	15.16	0.20	0.63	27.47	13.19	14.86	10.31	4.22	0.24
W10_8	8	10	2	1.83	1.47	3.52	3.93	0.66	1.44	1.22	13.51	0.13	0.66	24.92	17.38	9.84	14.01	5.29	0.20
W10_9	9	10	2	1.71	1.34	3.31	3.85	0.76	1.44	1.05	13.32	0.16	0.77	24.49	17.23	10.06	14.69	5.60	0.22
W10_10	10	10	2	1.61	1.83	3.72	2.97	0.98	1.52	1.24	13.38	0.19	1.06	21.19	19.01	7.67	17.13	6.29	0.23
W10_11	11	10	2	1.77	1.34	3.12	3.84	0.58	0.95	0.75	15.01	0.25	1.28	23.03	15.33	11.79	14.73	6.16	0.08
W10_12	12	10	2	2.01	1.50	3.30	3.84	0.74	1.47	0.43	14.84	0.33	1.57	20.67	15.52	9.78	16.39	7.35	0.28
W10_13	13	10	3	1.55	1.23	3.14	4.26	0.60	1.15	0.83	15.93	0.11	0.74	24.64	15.20	13.00	13.07	4.42	0.14
W10_14	14	10	3	1.66	0.97	2.94	4.44	0.11	0.97	0.43	13.99	0.16	0.97	27.76	14.08	16.11	10.75	4.65	0.01
W11_2	2	11	1	1.09	0.89	3.08	4.12	0.53	0.82	0.61	20.87	0.10	0.44	22.76	10.11	22.28	8.20	3.89	0.21
W11_3	3	11	1	1.38	1.10	3.30	4.03	0.76	1.10	0.91	15.66	0.20	0.83	23.00	13.75	15.22	12.59	5.89	0.29
W11_4	4	11	1	1.34	1.18	3.91	4.04	0.47	1.05	0.90	14.94	0.11	0.56	19.95	15.28	21.44	11.59	3.09	0.17
W11_5	5	11	1	1.55	1.19	3.20	4.14	0.80	1.11	1.01	14.06	0.23	0.95	24.97	14.93	12.91	12.97	5.71	0.29

W12_1	1	12	1	1.02	0.57	2.80	5.22	0.33	0.45	0.41	26.56	0.03	0.16	21.42	6.52	28.09	4.46	1.87	0.10
W12_2	2	12	1	1.68	0.90	3.46	5.64	0.57	0.77	0.77	18.52	0.05	0.29	26.34	11.17	17.94	8.79	2.98	0.13
W12_3	3	12	1	1.94	1.20	3.28	4.95	0.88	1.30	1.18	13.33	0.07	0.42	25.89	15.77	9.97	15.05	4.58	0.19
W12_4	4	12	1	1.70	1.03	3.48	5.52	0.54	1.09	0.90	16.20	0.04	0.25	27.03	13.90	15.38	10.23	2.59	0.11
W12_5	5	12	1	1.51	0.93	2.67	4.79	0.65	1.64	0.95	12.31	0.08	0.49	25.51	15.07	16.15	13.29	3.81	0.15
W12_6	6	12	1	1.54	1.03	3.02	3.87	0.75	1.56	1.14	12.36	0.05	0.37	24.02	18.06	11.76	16.29	4.05	0.13
W12_7	7	12	2	1.40	1.07	2.86	3.58	0.78	1.91	1.08	11.27	0.08	0.52	24.37	16.87	12.01	17.16	4.87	0.18
W12_8	8	12	2	1.85	1.05	3.27	4.30	0.73	1.93	1.24	13.41	0.09	0.52	24.13	16.67	11.49	15.27	3.84	0.20
W12_9	9	12	2	1.36	1.02	2.72	4.48	0.67	1.59	0.93	12.83	0.05	0.38	26.07	15.48	13.90	14.56	3.84	0.13
W12_11a	11	12	2	2.22	2.03	3.78	3.25	1.59	2.37	1.83	11.63	0.37	1.44	14.52	18.02	6.11	22.15	8.24	0.44
W12_11b	11	12	2	1.62	1.04	2.84	4.49	0.63	1.58	0.98	12.00	0.05	0.31	26.87	15.91	13.54	14.51	3.50	0.11
W12_12	12	12	2	1.92	1.56	3.59	4.23	1.25	1.87	1.42	13.97	0.22	1.06	20.95	15.33	11.01	15.81	5.45	0.37
W12_13	13	12	3	2.01	1.62	3.24	3.49	1.18	2.43	1.35	14.31	0.30	1.04	17.89	16.71	6.98	20.31	6.80	0.34
W12_14	14	12	3	1.59	1.29	3.10	3.63	0.85	2.34	1.21	13.66	0.15	0.72	21.64	18.03	9.40	17.00	5.16	0.22
W12_15	15	12	3	1.70	1.22	2.47	3.62	0.82	1.90	0.69	8.80	0.15	0.85	21.98	19.67	10.27	20.30	5.43	0.15
W12_16	16	12	3	1.65	1.19	2.26	3.42	0.76	2.46	0.76	8.25	0.19	0.93	21.70	19.61	8.56	22.80	5.29	0.17
W13_8	8	13	2	2.31	1.87	3.59	4.26	1.19	1.33	1.66	11.39	0.29	0.81	20.57	17.49	9.28	17.69	5.86	0.43
W13_10	10	13	2	2.16	2.36	3.72	2.90	1.59	0.50	1.54	9.16	0.16	0.81	19.02	20.98	5.28	23.11	6.44	0.26
W13_11	11	13	2	2.14	1.85	3.86	4.35	0.98	1.11	1.30	12.59	0.18	0.68	23.14	17.46	10.36	15.18	4.57	0.26
W13_12	12	13	2	2.45	1.83	4.06	4.56	0.82	1.22	1.45	13.84	0.00	0.44	22.52	18.00	9.52	16.15	2.89	0.25
W14_2	2	14	1	1.36	0.93	2.96	3.94	0.52	0.95	0.70	15.88	0.21	0.63	25.87	14.66	16.57	10.08	4.53	0.22

W14_3	3	14	1	1.30	0.83	2.23	3.73	0.49	1.04	0.81	11.30	0.07	0.35	27.09	16.55	14.33	15.29	4.47	0.14
W14_4	4	14	1	1.41	0.96	2.73	3.88	0.46	1.03	0.82	13.15	0.07	0.36	27.86	16.10	13.93	13.41	3.71	0.11
W14_5	5	14	1	1.42	0.83	3.07	4.23	0.37	0.79	0.69	16.97	0.07	0.37	28.95	14.32	14.18	10.51	3.12	0.10
W14_6	6	14	1	1.04	0.71	2.57	3.63	0.32	0.77	0.55	15.30	0.09	0.32	28.93	14.22	18.83	9.66	2.96	0.11
W14_7	7	14	2	1.43	1.02	3.30	3.94	0.48	0.86	0.78	15.41	0.08	0.36	29.26	14.92	12.97	12.16	2.93	0.09
W14_8	8	14	2	1.54	1.04	3.03	3.43	0.52	1.10	0.95	12.92	0.14	0.52	26.60	19.68	11.30	13.37	3.73	0.13
W14_10	10	14	2	1.44	0.79	2.62	4.57	0.41	0.86	0.76	14.69	0.11	0.50	27.16	14.28	18.07	10.67	2.97	0.11
W14_11	11	14	2	1.85	1.01	2.89	3.99	0.56	1.06	1.12	11.89	0.10	0.47	27.16	18.34	10.51	15.59	3.35	0.09
W14_12	12	14	2	1.33	0.82	2.52	4.13	0.45	0.98	0.78	13.16	0.11	0.44	26.28	15.49	16.71	13.18	3.50	0.11
W15_1	1	15	1	2.35	1.09	3.49	6.17	0.38	0.25	0.48	16.94	0.25	1.10	23.17	10.28	19.19	7.63	6.97	0.26
W15_2	2	15	1	1.98	1.00	3.13	6.57	0.51	0.47	0.30	16.69	0.18	0.87	25.79	10.25	20.03	6.75	5.24	0.26
W15_3	3	15	1	0.09	1.66	5.99	1.01	1.31	0.66	0.02	18.37	0.70	2.45	19.98	15.96	7.38	12.29	11.51	0.65
W15_4	4	15	1	2.07	1.34	3.68	5.49	0.52	0.40	0.28	16.33	0.15	0.75	26.40	13.48	14.41	9.75	4.73	0.21
W15_5	5	15	1	2.06	1.38	3.42	5.50	0.82	0.99	1.07	13.83	0.11	0.57	25.83	15.64	14.42	9.43	4.70	0.24
W15_6	6	15	1	2.27	1.12	3.16	6.98	0.65	0.74	0.95	12.89	0.10	0.53	27.27	13.43	16.50	9.56	3.63	0.21
W15_7a	7	15	2	2.08	1.45	2.72	5.33	0.83	0.51	0.03	11.27	0.24	1.05	25.17	15.24	15.15	12.95	5.73	0.24
W15_7b	7	15	2	2.15	1.79	3.23	3.91	1.27	1.52	1.53	9.47	0.14	0.89	21.71	19.16	8.19	17.65	7.07	0.33
W15_8a	8	15	2	1.93	1.33	2.90	5.35	0.89	0.56	0.78	11.57	0.26	1.05	23.56	15.26	14.76	14.09	5.48	0.23
W15_8b	8	15	2	2.28	2.32	3.81	4.10	2.11	1.88	2.05	9.21	0.73	2.39	15.16	15.97	5.51	20.30	11.27	0.89
W16_1	1	16	1	1.72	0.90	3.14	6.55	0.44	0.59	0.62	17.00	0.14	0.49	26.70	9.66	21.98	6.33	3.58	0.16
W16_2	2	16	1	1.74	1.02	2.96	5.44	0.74	1.12	0.92	10.91	0.33	1.24	26.67	13.06	15.29	11.27	7.00	0.31
W16_3	3	16	1	1.83	1.28	3.52	4.71	0.73	1.19	0.95	13.49	0.16	0.70	25.56	15.36	13.25	11.91	5.12	0.24

W16_4	4	16	1	1.84	1.14	3.55	5.98	0.71	1.06	0.88	14.30	0.19	0.75	25.52	12.91	16.22	10.48	4.28	0.19
W16_5	5	16	1	1.92	0.79	2.75	7.30	0.44	0.78	0.66	13.28	0.26	0.73	30.43	11.95	19.95	5.97	2.52	0.27
W16_7	7	16	2	1.51	1.70	4.03	3.60	1.27	1.37	1.17	11.95	0.47	2.00	18.82	14.21	10.95	16.75	9.71	0.49
W16_8	8	16	2	1.83	1.23	3.36	4.64	0.80	1.42	1.04	11.32	0.34	1.12	26.10	15.28	13.18	12.75	5.29	0.30
W17_0	0	17	1	1.80	1.09	3.52	5.28	0.67	0.45	0.97	17.44	0.21	0.84	20.68	9.01	20.41	6.51	10.53	0.61
W17_1	1	17	1	1.84	1.18	3.17	5.23	0.67	0.84	1.00	14.64	0.18	0.60	25.75	13.67	14.44	12.30	4.28	0.20
W17_2	2	17	1	2.51	1.56	3.37	5.78	0.92	1.06	1.47	10.32	0.07	0.42	27.65	14.88	10.80	15.29	3.79	0.12
W17_3a	3	17	1	2.34	1.29	2.92	5.67	0.82	0.94	1.19	7.92	0.11	0.48	28.79	16.89	12.45	14.54	3.54	0.12
W17_3b	3	17	1	2.31	1.01	1.94	4.71	0.54	0.94	0.77	5.00	0.11	0.69	28.44	19.96	10.07	18.46	5.04	0.02
W17_4a	4	17	1	1.70	1.62	2.43	2.83	1.27	1.69	1.55	6.19	0.21	0.83	19.93	19.89	6.20	26.40	7.07	0.21
W17_4b	4	17	1	2.46	1.31	3.36	4.52	0.69	0.73	0.72	9.54	0.05	0.38	31.14	18.52	9.73	13.29	3.46	0.10
W17_5	5	17	1	1.81	1.52	2.91	3.74	0.92	1.37	1.29	8.92	0.12	0.63	24.06	19.12	9.15	19.52	4.79	0.12
W18_36	36	18	4	1.53	1.35	3.14	3.91	0.74	0.33	0.51	12.53	0.16	0.56	26.16	15.39	15.05	13.27	5.18	0.19
W19_35	35	19	4	1.69	2.03	3.12	2.56	1.55	0.66	1.12	8.84	0.17	1.16	18.50	18.67	6.56	22.22	10.83	0.33
W20_29	29	20	4	1.93	1.71	3.60	4.13	1.02	1.87	0.91	12.63	0.21	1.01	22.89	17.42	8.27	16.41	5.71	0.27
W20_30	30	20	4	1.68	1.61	3.41	4.07	0.95	1.71	1.00	14.31	0.19	1.11	21.25	15.98	9.18	17.20	6.09	0.25
W21_29	29	21	4	1.47	1.70	2.91	2.50	1.61	1.11	1.26	10.04	0.33	2.18	15.93	15.94	5.07	25.44	12.20	0.33
W22_28	28	22	4	2.03	1.59	2.96	3.77	0.89	1.21	1.31	10.37	0.21	1.15	20.25	18.73	8.05	21.25	6.06	0.16
W22_29	29	22	4	1.59	1.58	2.97	4.31	1.09	1.67	1.23	12.75	0.16	1.10	20.53	17.98	10.90	17.33	4.66	0.16
W22_30	30	22	4	1.87	1.70	3.04	3.67	1.06	1.16	1.18	10.96	0.08	0.62	22.44	18.93	8.17	20.91	4.10	0.12
W23_26	26	23	4	1.31	1.78	2.81	2.05	1.38	0.87	1.01	8.31	0.21	1.35	18.93	19.05	6.51	24.41	9.69	0.33

W24_0	0	24	1	1.62	0.89	5.74	5.48	0.24	0.11	0.01	38.63	0.00	0.48	14.65	4.98	12.07	2.35	12.29	0.46
W25_0	0	25	1	1.54	1.21	3.72	4.01	0.21	0.66	0.08	24.18	0.02	0.77	20.87	12.68	18.06	6.84	4.57	0.57
W26_0	0	26	1	0.07	1.01	0.81	4.22	0.31	0.12	0.03	24.00	0.04	1.12	19.65	8.26	28.00	5.00	7.11	0.27
W27_0	0	27	1	1.10	0.88	4.77	3.95	0.55	0.33	0.11	35.69	0.02	0.78	12.56	4.79	11.72	2.86	19.23	0.66
W28_0	0	28	1	2.02	1.55	4.12	4.16	0.52	0.03	0.02	16.85	0.44	1.56	17.53	10.09	13.31	9.17	17.56	1.06
W29_0	0	29	1	1.59	0.97	3.67	4.63	0.49	0.00	0.35	26.65	0.00	0.48	20.45	9.44	21.41	4.84	4.66	0.38

Table S4. Average percent relative distribution of GM3 species throughout the lactation

Lactation month	0 n=7	1 n=13	2 n=15	3 n=16	4 n=16	5 n=15	6 n=11	7 n=13	8 n=12	9 n=8	10 n=6	11 n=10	12 n=9	13 n=4	14 n=4	15 n=3	16-19 n=5	> 24 n=9
GM3 d42:2	26.21	17.46	13.89	13.22	13.22	13.22	13.29	12.25	11.72	12.43	11.95	11.94	12.13	14.65	12.89	10.78	14.20	11.03
GM3 d40:1	18.05	25.46	25.82	26.16	26.05	25.92	24.66	25.26	24.31	26.80	24.08	23.97	22.97	21.96	24.84	22.83	24.72	20.09
GM3 d42:1	17.86	19.69	15.40	13.07	13.93	12.93	11.87	12.69	11.11	12.00	10.90	9.82	10.09	9.23	13.54	10.32	13.83	7.84
GM3 d34:1	10.85	4.44	5.19	5.22	4.45	4.86	5.44	5.14	5.12	4.10	4.77	4.98	5.17	6.22	5.09	5.73	4.28	7.42
GM3 d38:1	8.46	10.79	13.67	14.97	15.43	15.86	15.70	16.36	17.23	17.39	17.56	17.95	17.18	16.34	15.91	17.53	15.81	17.84
GM3 d36:1	5.37	8.39	11.93	13.03	12.86	12.82	15.00	14.24	15.47	14.16	16.99	17.14	17.96	17.37	14.47	19.35	14.86	20.65
GM3 d41:1	4.53	5.16	4.74	4.61	4.48	4.47	3.81	4.14	4.08	3.74	3.66	3.66	3.71	3.38	4.06	3.56	3.94	3.38
GM3 d40:2	3.76	3.19	3.08	3.25	3.20	3.16	3.25	3.07	3.15	2.95	2.99	3.12	3.09	3.18	2.79	2.41	2.44	3.10
GM3 d39:1	1.61	1.53	1.66	1.68	1.70	1.74	1.55	1.69	1.82	1.62	1.58	1.72	1.72	1.62	1.58	1.55	1.43	1.70
GM3 d38:2	1.09	1.01	1.13	1.19	1.18	1.19	1.23	1.24	1.35	1.14	1.36	1.32	1.35	1.33	1.13	1.17	0.98	1.71
GM3 d32:1	0.86	0.53	0.64	0.66	0.56	0.66	0.66	0.71	0.83	0.52	0.69	0.76	0.80	1.04	0.80	0.97	0.83	1.21
GM3 34:2	0.57	0.23	0.22	0.21	0.21	0.22	0.25	0.21	0.26	0.15	0.17	0.18	0.21	0.25	0.14	0.17	0.13	0.24
GM3 d36:2	0.43	0.57	0.70	0.73	0.63	0.69	0.80	0.73	0.84	0.63	0.82	0.83	0.87	0.84	0.61	0.78	0.56	1.19
GM3 d37:1	0.26	0.73	0.87	0.85	0.90	0.96	1.02	0.94	1.18	0.97	1.13	1.09	1.09	0.96	0.81	0.91	0.65	1.13
GM3 d36:0	0.24	0.67	0.91	1.00	1.09	1.17	1.34	1.17	1.32	1.29	1.20	1.36	1.52	1.42	1.19	1.79	1.20	1.28

GM3 30:1	0.10	0.13	0.13	0.14	0.12	0.15	0.13	0.15	0.21	0.10	0.15	0.15	0.14	0.20	0.15	0.16	0.15	0.20

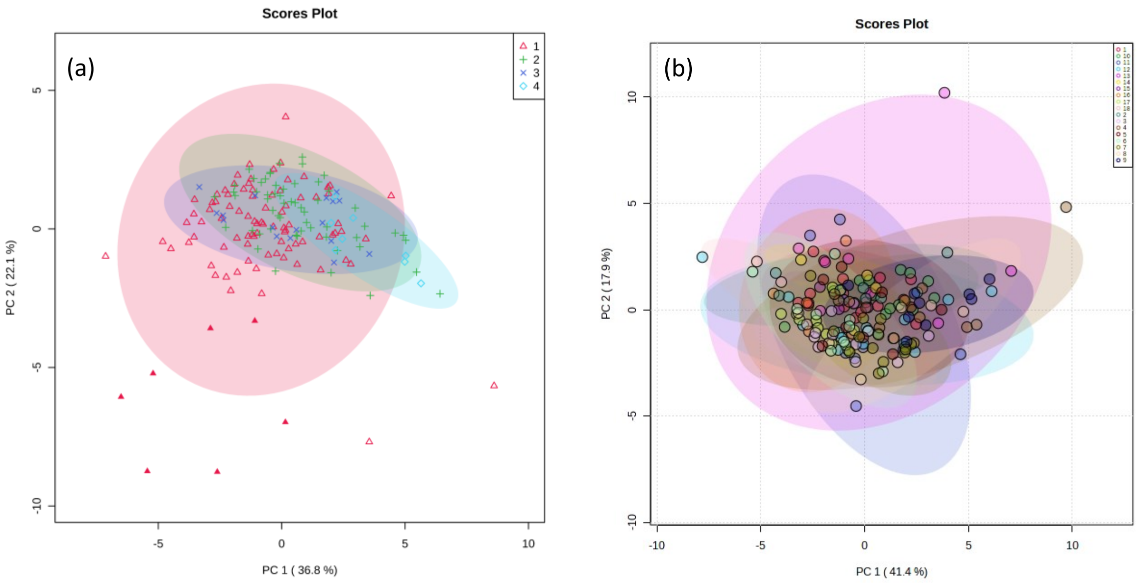

Figure S4. PCA score plot of GM3 profiles of human milk samples collected at different lactation point. The groups are defined based on lactation period (a) or HM donor (b). Colostrum samples are marked as red filled triangles.

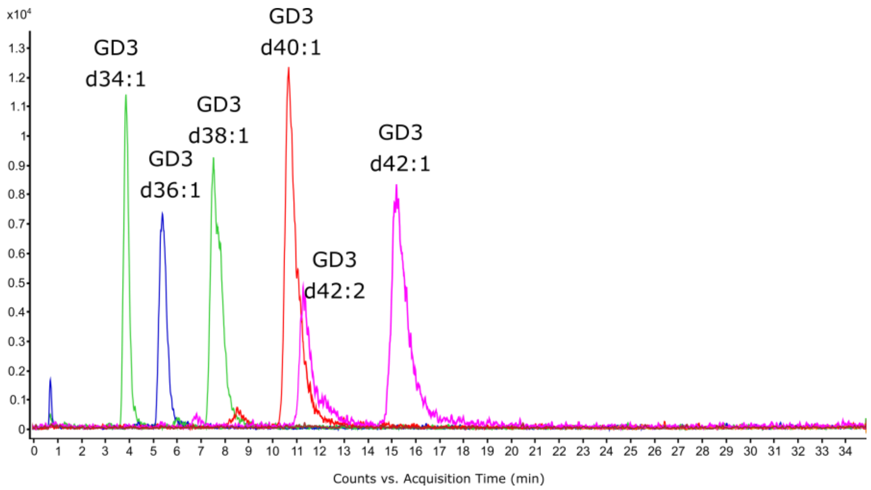

Figure S5. Exemplary extracted ion chromatograms of the GD3 species analyzed in colostrum sample with the use of RP-LC-Q-TOF-MS technique in negative ionization mode.
